# mTOR-regulated *U2af1* tandem exon splicing specifies transcriptome features for translational control

**DOI:** 10.1093/nar/gkz761

**Published:** 2019-09-03

**Authors:** Jae-Woong Chang, Hsin-Sung Yeh, Meeyeon Park, Luke Erber, Jiao Sun, Sze Cheng, Alexander M Bui, Naima Ahmed Fahmi, Ryan Nasti, Rui Kuang, Yue Chen, Wei Zhang, Jeongsik Yong

**Affiliations:** 1 Department of Biochemistry, Molecular Biology and Biophysics, University of Minnesota Twin Cities, Minneapolis, MN 55455, USA; 2 Department of Computer Science, University of Central Florida, Orlando, FL 32816, USA; 3 Department of Genetics, Cell and Developmental Biology, University of Minnesota Twin Cities, Minneapolis, MN 55455, USA; 4 Department of Computer Science and Engineering, University of Minnesota Twin Cities, Minneapolis, MN 55455, USA

## Abstract

U2 auxiliary factor 1 (U2AF1) functions in 3′-splice site selection during pre-mRNA processing. Alternative usage of duplicated tandem exons in *U2AF1* produces two isoforms, U2AF1a and U2AF1b, but their functional differences are unappreciated due to their homology. Through integrative approaches of genome editing, customized-transcriptome profiling and crosslinking-mediated interactome analyses, we discovered that the expression of U2AF1 isoforms is controlled by mTOR and they exhibit a distinctive molecular profile for the splice site and protein interactomes. Mechanistic dissection of mutually exclusive alternative splicing events revealed that U2AF1 isoforms’ inherent differential preferences of nucleotide sequences and their stoichiometry determine the 3′-splice site. Importantly, U2AF1a-driven transcriptomes feature alternative splicing events in the 5′-untranslated region (5′-UTR) that are favorable for translation. These findings unveil distinct roles of duplicated tandem exon-derived U2AF1 isoforms in the regulation of the transcriptome and suggest U2AF1a-driven 5′-UTR alternative splicing as a molecular mechanism of mTOR-regulated translational control.

## INTRODUCTION

Eukaryotic pre-mRNA is spliced to mRNA by the spliceosome which is composed of small nuclear ribonucleoprotein complexes (snRNPs). Among those snRNPs in the spliceosome, U2 snRNP is critical for splicing by recognizing the branch point (1). U2 auxiliary factors (U2AFs) are known to bind to polypyrimidine tracts near 3′-splice sites and recruit U2 snRNP to the branch point. U2AFs are a heterodimer consisting of U2AF1 (formerly known as U2AF35) and U2AF2 (formerly known as U2AF65). U2AF2 recognizes the polypyrimidine tract while U2AF1 is known to bind to the AG dinucleotide at 3′-splice site (2–6).

The U2AF1 gene contains duplicated tandem exons between exon 2 and exon 4. These two duplicated tandem exons (3a and 3b (formerly designated as exon Ab)) are mutually exclusive in splicing and yield two highly similar isoforms, U2AF1a and U2AF1b. They are evolutionary conserved and only differ by seven amino acids in the final protein products (97.1% identity) (7,8). It has been shown that U2AF1a is more abundant than U2AF1b in various cell lines and tissues (7–9). Because of inherent similarities and biased expression of U2AF1a, studies on the functional differences between U2AF1 isoforms are largely lacking. Other than a few examples, functional differences between tandem exon-derived isoforms are not well characterized due to similar reasons. However, examples of *PKM* and *FGFR2* genes provide evidence that tandem exon-derived isoforms function differently and distinctively affect cells (10–13).

It is known that three transcripts are transcribed from the *U2AF1* gene. Evolutionarily conserved, mutually exclusive tandem exons drive the transcription of the two isoform transcripts, *U2AF1a* and *U2AF1b*, and the inclusion of both exons produce *U2AF1c* transcript which is subjected to nonsense-mediated mRNA decay (8). The differences between two U2AF1 isoforms encoded by alternative exon 3 usage occur at the atypical RNA recognition motif which is involved in the dimerization with U2AF2 (14). Regardless of the seven amino acid differences, however, the two U2AF1 isoforms have biochemically been shown to be similar in forming U2AF heterodimers and functioning in pre-mRNA splicing (8).

The mammalian target of rapamycin (mTOR) pathway has pivotal roles in cell growth, protein translation, and survival (15). Tuberous sclerosis complexes (TSC1 and TSC2) negatively regulate mTORC1 kinase and genetic knockdown or knockout of TSC (*Tsc1^−/−^* or *Tsc2^−/−^*) hyperactivates mTORC1 (16,17). We previously showed that mTOR is involved in alternative polyadenylation (APA) and promotes transcriptome-wide APA in 3′-untranslated regions (3′-UTRs), affecting diverse cellular pathways including ubiquitin-mediated proteolysis and ER stress responses (18,19). These studies suggested that mTOR may function in the processing of pre-mRNA in addition to well-characterized roles in various cellular pathways.

In this study, we investigated the transcriptome changes upon mTOR activation and found that the stoichiometry of U2AF1 isoforms is drastically altered by cellular mTOR activity. We further delineated the functional differences of duplicated tandem exon-derived U2AF1 isoforms by taking integrative approaches of genome-editing and high profiling methodologies. Unlike previous suggestions (9), our unbiased approaches revealed that U2AF1 isoforms contribute differentially to transcriptome changes by alternative splicing and affect protein synthesis by regulating 5′-UTR alternative splicing.

## MATERIALS AND METHODS

### Cell lines and cell culture

WT MEF and *Tsc1^−/−^* MEF cells were obtained from Dr Kwiakowski at Harvard University and they were previously described (16,18,19). WT, *Tsc1^−/−^* MEF, HEK293, HeLa and MDA-MB231 cells were cultured in Dulbecco's Modified Eagle Medium (Gibco, USA) with 10% (v/v) fetal bovine serum (FBS) and 100 g/ml streptomycin and 100 U/ml penicillin at 37°C in 5% CO_2_.

### Construction of CRISPR/Cas9-sgRNA plasmids for U2AF1 tandem exon knockout

The target gRNA sequences were identified by crispor.tefor.net and chopchop.rc.fas.harvard.edu. The guide sequences were cloned into Addgene plasmid #48138 using the following primers. Targeting exon 3b, 5′ end forward 5′-CACCGTTGAATCAAGATGGTCTGCG-3′reverse 5′-AAACCGCAGACCATCTTGATTCAAC-3′; 3′ end forward 5′-CACCGCACACTGTAAGTCCCACAGT-3′ reverse 5′-AAACACTGTGGGACTTACAGTGTGC-3′. Targeting exon 3a #1, 5′ end forward 5′-CACCGAGAGGTGTCCCCTTAGTTGG-3′ reverse 5′-AAACCCAACTAAGGGGACACCTCTG-3′; 3′ end forward 5′-CACCGAGTTCAGATCTCGAGGTGAG reverse 5′-AAACCTCACCTCGAGATCTGAACTC-3′. Targeting exon 3a #2, 5′ end forward 5′-CACCGCTGGGCTGGCACTTAGCAG-3′ reverse 5′-AAACCTGCTAAGTGCCAGCCCAGC-3′; 3′ end forward 5′-CACCGGGCAGGAGTTCAGATCTCG-3′ reverse 5′-AAACCGAGATCTGAACTCCTGCCC-3′.

### RNA sequencing and analyses

poly(A+) RNAs were isolated from U2af1**a**- and U2af1**b**-only#1 cell lines treated with control or *U2af1* targeting siRNA were sent out for paired end reads RNA-seq analysis. A total of 84 452 901 reads for U2af1**a** only#1 control cells, 91 886 993 reads for U2af1**a** only#1 siRNA cells, 84 722 415 reads for U2af1**b** only#1 control cells, and 86 708 880 reads for U2af1**b** only#1 siRNA cells were produced from Hi-Seq pipeline with length of 51 bp of each end. The short reads were aligned to mm10 reference genome by TopHat, with up to two mismatches allowed. 93.4% of paired short reads from U2af1**a** only#1 control, 93.5% reads from U2af1**a** only#1 siRNA, 93.0% reads from U2af1**b** only#1 control, and 93.9% reads from U2af1**b** only#1 siRNA were mapped to the reference genome for alternative splicing analysis in the study.

### AS-Quant for the analyses of alternative splicing events

AS-Quant first applies rMATS (20) to categorize potential alternative splicing events into four categories (cassette type, SE; mutually exclusive, MXE; alternative 5′-splice site, A5SS; alternative 3′-splice site, A3SS) based on the UCSC annotation. Then for each categorized potential alternative splicing event, the mean short read coverages of the affected exon and the rest of exons in the transcript are measured, and we denote them as *n* and *N* based on the above context using RNA-seq alignment file. Next, a canonical 2 × 2 Chi-squared test is applied to report a *P*-value for each candidate event based on the *n*/*N* ratios in two cases. The candidate alternative splicing events with *P*-value ≤0.1 and the ratio difference larger than 0.1 between the two cases are considered for further analyses.

### Western blotting

Antibodies used in this study include: anti-U2AF1 (ab86305, Abcam), anti-U2AF2 (sc-48804, Santa Cruz Biotechnology), anti-hnRNP A1 (4B10, Abcam), anti-RPS6 (#2317, Cell Signaling), and anti-pRPS6 (#2211, Cell Signaling), anti-TUBULIN (sc-53646, Santa Cruz Biotechnology), anti-SRSF3 (sc-13510, Santa Cruz Biotechnology), anti-EIF4EBP1 (#9452, Cell Signaling), anti-HNRNPC1/C2 (ab10294, Abcam), anti-Flag (F3165, Sigma Aldrich).

### Minigene reporter assay

Minigene U2af1 reporter gene fragment was amplified at genomic region from the start of exon 2 to the end of exon 4 with forward primer 5′-GCCATGGATCCAGTCAACTGTTCATTTTATTTC-3′ and reverse primer 5′-.ATATTAGAGCGGCCGCCTCAAAGAACTCATCATAG-3′. The fragments were then digested with BamHI and NotI, then ligated into pcDNA3.1 (+) plasmid (Thermo Fisher Scientific).

### CRISPR-induced homologous recombination

To insert a 3x-Flag tag at the C-terminal of *U2af1* gene via homologous recombination, the donor sequence was synthesized as a gBlocks gene fragment (IDT) and cloned into pAAV-SEPT-acceptor vector (Addgene). Type IIS restriction enzyme BspQI was used for the cloning. To induce efficient homologous recombination near the C-terminal locus of *U2af1*, a double-stranded break was created by CRISPR/Cas9 cloned into PX458 (GFP+) using primer sequences as follows: forward 5′-CACCGACACACGGTAAAAAGGGCT-3′ reverse 5′-AAACAGCCCTTTTTACCGTGTGTC. The two plasmids were co-transfected into U2af1**a**-only#1 and U2af1**b**-only#1 cell lines. Top 20% GFP+ cells were isolated for further screening by flow cytometry. Edited clones were confirmed by PCR of genomic DNA and Western blot analysis.

### Immunoprecipitation and mass spectrometry

Cells were fixed and crosslinked with 0.2% formaldehyde for 10 min at room temperature and quenched with 0.15 M glycine pH 7.5, then washed with PBS twice. The pellet was resuspended with lysis buffer (25 mM Tris pH 7.4, 300 mM NaCl, 2.5 mM MgCl_2_, 0.5% Empigen; 0.5% NP-40) and sonicated with microtip for four times, 10 s each at 4 W, and spun down at maximum speed for 10 min. The supernatants were incubated with anti-Flag M2 magnetic beads (Sigma-Aldrich, M8823) in an end-over-end rotator at 4°C for 2 h. The beads were washed five times with lysis buffer. The protein complexes were eluted with 5 volumes of beads with 3× flag peptide (150 ng/ul). The elutions were precipitated with acetone and resuspended with 5× SDS sample buffer by boiling for 15 min. Samples were run on a 10% criterion gel. The gel was fixed with 40% ethanol and 10% AcOH and washed with ddH_2_O. Lastly, the gel was stained with imperial stain and the stained areas were cut out for in-gel trypsin digestion. The gel pieces were washed with 50% ethanol twice for 2 and 16 h with mixing. The gel pieces were washed twice with water for 10 min with mixing and then cut into mm^3^ size pieces. After drying with 100% acetonitrile, the gel pellets were reduced and alkylated with 10 mM tris(2-carboxyethyl)phosphine and 40 mM iodoacetamide respectively. The gel was washed with 50% acetonitrile and 50 mM ammonium bicarbonate for 5 min and dried in speedvac. The gel pieces were digested with 0.1 ug Trypsin overnight at 37°C with rotation and extracted twice with 50% acetonitrile with 1% TFA and 100% acetonitrile. The peptide solution was dried with speedvac and desalted with C18.

### LC–MS/MS measurement

LC–MS/MS analysis was performed using a Proxeon Easy nLC 1000 Nano-UPLC system coupled with an Orbitrap Fusion mass spectrometer (ThermoFisher). Peptide samples were loaded onto custom packed C18 column (15 cm × 75 μm, ReproSil-Pur Basic C18, 2.5 μm, Dr Maisch GmbH) and eluted for 2 h using a 5–32% gradient of HPLC solvent B (0.1% formic acid in acetonitrile, v/v) and a flow rate of 200 nl/min. Fusion Orbitrap was operated in data-dependent mode. Survey scan MS were acquired with the orbitrap with a 380–1580 *m*/*z* range and a resolution of 60 000. Ions were selected by using dynamic exclusion of 15 s, an intensity threshold of 1.0E4 and charge states of 2–7. The top 12 most intense ions per survey were selected for CID fragmentation and ion trap analysis

### Raw mass spectrometry data processing

Raw mass spectrometry files were processed by MaxQuant (version 1.5.3.12) for database search and quantitative analysis. Cysteine carbamidomethylation was set as a fixed modification and methionine oxidation and protein N-terminal acetylation were set as variable modifications. The proteolytic enzyme was set as trypsin with a maximum of two missing cleavages. The data was searched against the UniProt mouse database (downloaded at 27 September 2013 with 43 310 sequences), and we used a cutoff threshold setting at 1% false discovery rate (FDR) at protein and peptide levels. The precursor ion tolerance was set to 4.5 ppm and the fragment ion mass tolerance was set to 0.5 Da. The MaxLFQ algorithm provided by MaxQuant was selected for the label-free relative quantification of the samples. To perform relative quantification, the LFQ metrics were extracted from the MaxQuant-processed data and processed for statistical analysis using the Perseus software (version 1.5.5.1). Multiple hypothesis testing was performed using two-sided Student's *t*-test and permutation-based FDR correction. The FDR was set at 5% and the S0 variance correction constant was set at 0.1 for all comparisons.

### Co-immunoprecipitation

RSB-100 buffer (25 mM Tris, pH 7.4; 100 mM NaCl, 2.5 mM MgCl_2_; 0.02% Triton-X-100) was used as binding buffer for co-immunoprecipitation experiments. Nuclear extract of HEK293 cell line was prepared according to the REAP method (21). Nuclear pellet was resuspended with RSB-100 and sonicated twice, 5 s each at 1 W. After 30 s spin-down at 8000g, the nuclear extract was incubated with protein G sepharose beads immobilized with anti-HNRNPA1, anti-HNRNPC1/C2 or anti-Flag antibodies for 1 h at 4° on an end-over-end rotator. Beads were then washed three times. Elution was done by adding 4× SDS sample buffer to the beads followed by 10 min-boiling. Elutions were run on 12.5% SDS-PAGE for western blot analysis.

### qPCR and RT-PCR

Total RNAs were isolated using Trizol reagents according to manufacturer's protocol. The extracted RNAs were reverse transcribed into cDNA using oligo-d(T) or random hexamer priming and superscript III (Thermo Fisher Scientific) from standard protocol supplied by the manufacturer. For qPCR, cDNA templates were amplified and the Ct values were quantified in real time using Eva Green or Taqman probes where indicated. Normalization of the Ct values were performed for relative quantitation. Absolute quantitation was made where indicated. Primers and Taqman probes used in qPCR assays include: *U2af1a* Taqman probe, 5′-(FAM)- TTTAGCCAGACCATTGCCCTCTTGA –(BHQ-1)-3′. *U2af1a* forward primer, 5′- ATGGCGGAATACTTGGCCTC-3′; reverse primer, 5′-GTCAGCAGACTGGGAAGAGT-3′; *U2af1b* Taqman probe, 5′-(FAM)- ACGGCTCACACTGTGCTGTGAGCGA-(BHQ-1)-3′. *U2af1b* forward primer, 5′-ATCGTAATCCCCAAAACAGTGC-3′; reverse primer, 5′-AGACTTCCTCAAAGAACTCATCAT -3; *Anapc10* forward 5′AAGCAGTTGGAGAGGACAGC-3′ reverse 5′-ACCCTGGTTTGCAGGAAGAG-3′; *Hnrnph2* forward 5′-CACAGGGGAAGCTTTTGTGC-3′ reverse 5′-GGACTTCAGCTCGGCTACTC-3′; *Srsf1* forward 5′- ATCTCACGAGGGAGAAACTGC-3′ reverse 5′- GTAACTGCGACTCCTGCTGT -3′; *Srsf2* forward 5′-GCCCGAAGATCCAAGTCCAA-3′ reverse 5′- TGGACTCTCGCTTCGACAC-3′; *Srsf3* forward 5′-GCTGCCGTGTAAGAGTGGAA-3′ reverse 5′- AGGACTCCTCCTGCGGTAAT-3′; *Srsf4* forward 5′- AGCCGCAGTAAGAAGGAGAAA-3′ reverse 5′-GTCCTCGGCGTGGTCTTTA -3′; *Srsf5* forward 5′-AGGTCAAGAAGCAGGTCACG-3′ reverse 5′- TCGGCTGTAAGACTTGCTCC-3′;*Srsf6* forward 5′-GTCTCGGAGCAAAGGTCGAT-3′ reverse 5′-CTTGAGTGGGAATGGGAGCC-3′; *Srsf7* forward 5′- TGCAGAGGATGCAGTTCGAG-3′ reverse 5′-GGGCAGGTGGCCTATCAAAA-3′;*Srsf9* forward 5′-TCACGAGGGTGAGACTTCCT-3′ reverse 5′-GACCGCGACCGTGAGTAG-3′; *Srsf10* forward 5′-TCTCGAAGCCGGAGTTATGA-3′ reverse 5′-AGTCGGTCTACTGTTTCTAGGACT-3′; *Srsf11* forward 5′- GATCTCGCTCGAGGAGGAGG -3′ reverse 5′-TGGATTTGGAGTGTGACCGC -3′; *Srsf12* forward 5′-GAAATCACAGTCACGCTCGC-3′ reverse 5′- CTCTGGGAGACTTGCATGGG-3′; *Cpsf1* forward 5′-ACATACCGACGCTTGCTGAT-3′ reverse 5′-TAGCGGTTTAGCAGTTCCCC-3′; *Cpsf2* forward 5′- CGGAATTTGTAGGGGGCGTA-3′ reverse 5′-ATCCGATGCGTCCAGTTTCT-3′; *Cpsf3* forward 5′-GCACGTTTACAGCAAGAGGC-3′ reverse 5′- TTCTACAGCCCGAGTCTCCA-3′; *Cpsf4* forward 5′-GCACCCTCGATTTGAACTGC-3′ reverse 5′-CTGCATGACCCCAATGACCT-3′; *Cpsf5* forward 5′-AAGCCTTGTTTGCAGTCCCTA-3′ reverse 5′-AATGATGGGTCCATACCCCG-3′; *Cpsf6* forward 5′-TCACGGGAAAAGAGTCGTCG-3′ reverse 5′- CGGTATTCTCGCTCTCGGTC-3′; *Cpsf7* forward 5′- TGATTCTGCTGATGGACGGG-3′ reverse 5′-GGCAGACCCATTAGGGGAAG-3′.

For RT-PCR, cDNAs were amplified using primer sets listed below and the PCR products were subjected to electrophoresis in 2% agarose gel with ethidium bromide for visualization of amplified DNA fragments. Primer sets used in RT-PCR assays are as follows:

*Anapc10* forward 5′-GAACCGGAATTGTGGCGAATC-3′ reverse 5′-GGAGGTGTCTTGTTCGGTGT-3′; *Anapc10* alternative tss forward 5′-GCTGTCCTCTCCAACTGCTT-3′ reverse 5′-TGCTGTCTCCTCAGGCTTTG-3′; *Hnrnph2* forward 5′-GGTCGTCGTCTATCGTCTCG-3′ reverse 5′-AGCTTGGCTCAATGCAAATTC-3′; *Serpinh1* forward 5′-CTGTCTGAGGAGCGATTGCC-3′ reverse 5′-CAAGAGGCATAAGGTGCCCA-3′; *Gng12* forward 5′-GGGAAGGACTTTGGGGTGAG-3′ reverse 5′-CTATGCTGTTGGTGCTTGCC-3′; *Pcbp2* forward 5′-TTGACCAAGCTGCACCAGTT-3′ reverse 5′-TTGATTTTGGCGCCTTGACG-3′; *Ktn1* forward 5′-AGCTGACGAGTCTCAAAGGA-3′ reverse 5′-CACGTAAGTCGATCGCTCCAT-3′; *Tial1* forward 5′-TCAGTCAGATCGGACCCTGT-3′ reverse 5′-AGCAGCTGCATCTCTGTGTT-3′; *Puf60* forward 5′-TGCAATGGAGCAGAGCATCA-3′ reverse 5′-ATGCTCTTGATGGGGCCAAA-3′; *Pex2* forward 5′-ATGTCCACAGGATCCATGCC-3′ reverse 5′-TGGCTCAAAGCGAGCTAACA-3′.

### Polysome fractionation

Isolation of polysome fractions from total cell lysates using sucrose gradient was carried out as previously described (18,19). Briefly, cells were lysed in the polysome buffer (20 mM Tris pH 7.4, 150 mM NaCl, 5 mM MgCl_2_, 1 mM dithiothreitol, 100 mg/ml cycloheximide and 1% Triton X-100). Cell extracts were loaded onto sucrose gradient (5–45%). Fractionation was done by centrifugation at 190 000 × g for 2 h at 4°C. Twelve fractions were collected for the analysis. Amounts of mRNAs in each fraction were calculated using absolute quantitation. Ten per cent (v/v) of total RNAs in each fraction were used for RT-qPCR.

### Luciferase construct and assay

5′-UTRs with or without the alternative exons of *Hnrnph2, Anapc10, Pex2* and *Cwc22* were cloned into psiCheck1 (kindly provided by Dr Aaron Goldstrohm at the University of Minnesota Twin Cities; (22)). These recombinant luciferase reporters were transfected into *Tsc1^−/−^* MEFs using Lipofectamine 3000 (Thermo Fisher Scientific). Eighteen hours after transfection, the luciferase activity was measured using Dual-Glo reagent with the Glomax Discover luminometer (Promega). Expression of Renilla luciferase mRNA was measured to normalize the luciferase activity by RT-qPCR using the following primers: forward 5′-TCTCGTTAAGGGAGGCAAGC-3′ reverse 5′-TGGAAAAGAACCCAGGGTCG-3′. Four replicates of the measurement were conducted for technical repeats.

## RESULTS

### Mutually exclusive expression of *U2af1* isoforms is associated with cellular mTOR activity

**Figure 1.**
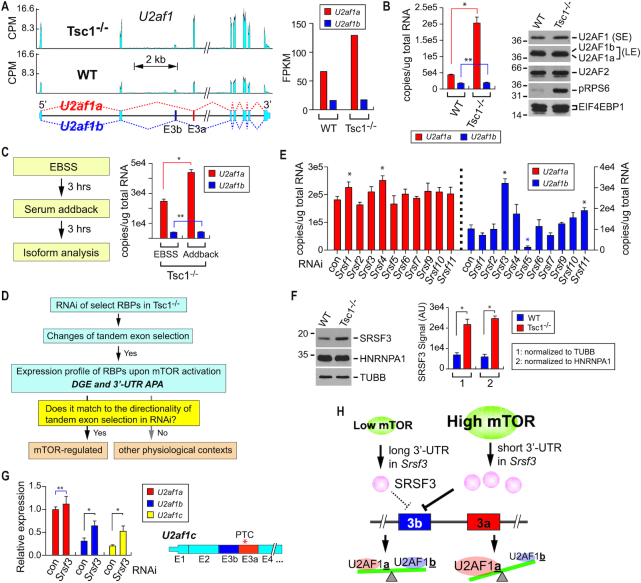
Cellular mTOR activity affects the expression profile of *U2af1* isoform. (**A**) *U2af1a* is selectively up-regulated upon mTOR activation. (left) RNA-Seq reads alignments of *U2af1* isoforms in WT and *Tsc1^−/−^* transcriptomes. (right) Quantitation of *U2af1* isoforms in the RNA-Seq data from WT and *Tsc1^−/−^* MEFs. (**B**) Expression of *U2af1* isoforms in WT and *Tsc1^−/−^* MEFs was measured by Taqman qPCR with absolute quantitation. The data are presented as the mean (SD) (*P = 1.3e-6, **P = 0.60; two-tailed Student's *t* test, *n* = 3 for technical repeats). Western blot analyses of U2AF1 isoforms were done using total cellular extracts from WT and *Tsc1^−/−^* MEFs. Please note that U2AF1b isoform is only visible in the longer exposure blot. Phospho-S6 (pRPS6) probing is for the validation of mTOR activation. SE and LE indicate short exposure and long exposure in western blot, respectively. (**C**) *U2af1a* is selectively up-regulated upon the activation of cellular mTOR signaling. (left) A workflow of the serum add-back experiment for the manipulation of cellular mTOR activity. (right) Absolute quantitation of *U2af1* isoforms by Taqman qPCR. The data are presented as the mean (SD) (*P = 1.6e–4, **P = 0.43; two-tailed Student's *t* test, *n* = 3 for technical repeats). (**D**) A workflow of the screening strategy for mTOR-regulated splicing factors that regulate the *U2af1* isoform expression. DGE, differential gene expression at the transcript level. (**E**) An RNAi screen to identify a regulator(s) of *U2af1* alternative splicing. SR splicing factors were knocked down by siRNAs in *Tsc1^−/−^* MEFs and the expression of *U2af1* isoforms was measured by Taqman qPCR assay with absolute quantitation. Asterisks denote statistically significant changes of *U2af1* isoform expression upon the RNAi knockdown. The data are presented as the mean (SD) (*P < 0.0086; two-tailed Student's *t* test, *n* = 3 for technical repeats). (**F**) Western blot analysis of SRSF3 in WT and *Tsc1^−/−^* MEFs. TUBULIN and HNRNPA1 were used as loading controls. Quantitation by ImageQuant software of the SRSF3 signals normalized to TUBULIN or HNRNPA1 is shown on the right. (*P < 7.5e-4; two-tailed Student's *t* test, *n* = 3 for biological repeats; see Supplementary Figure S1N for the other two repeats). (**G**) Relative expression of *U2af1a, U2af1b* and *U2af1c* transcripts (structure shown on right; PTC, premature termination codon) upon RNAi knockdown of *Srsf3*. Puromycin was added for 8 h at the concentration of 5 μg/ml. The data are presented as the mean (SD) (*P <0.010, **P = 0.31; two-tailed Student's *t* test, *n* = 3 for technical repeats). (**H**) A proposed model for regulation of *U2af1* tandem exon splicing by mTOR and SRSF3.

To better understand how mTOR contributes to transcriptome changes, we analyzed our previous RNA-Seq data from WT and *Tsc1^−/−^* MEFs (19) by focusing on the changes of gene expression in RNA-binding proteins (RBPs). Among the RBPs whose transcript levels changed upon mTOR activation, *U2af1* was particularly interesting: one of the two *U2af1* isoforms, *U2af1a* (uc008bvo.2, NM_024187.4), shows a ∼2-fold expression difference in *Tsc1*^−/−^ MEFs while the *U2af1b* (uc012aov.1, NM_001163769.1) expression remained unchanged between WT and *Tsc1^−/−^* MEFs (Figure 1A and B, Supplementary Figure S1A–D). This observation suggests that the biased expression of *U2af1a* isoform is associated with cellular mTOR signaling. To test this idea, we incubated *Tsc1*^−/−^ MEFs in Earle's balanced salt solution (EBSS) to reduce the cellular mTOR activity, followed by re-activation of the mTOR activity by incubating cells in serum-containing Dulbecco's modified eagle media (DMEM) (Figure 1C, Supplementary Figure S1E). In this experiment, while the level of *U2af1b* transcript remained unchanged, the expression of *U2af1a* was selectively increased (Figure 1C). Together, these data show that the mTOR signaling pathway regulates the mutually exclusive alternative splicing of duplicated tandem exons in *U2AF1* expression.

*U2AF1a*-polarized expression has been found in tissues and cell lines previously (8) and a weak branch point consensus sequence upstream of exon 3b has been proposed to be the reason for this observation (23). If this is the case, however, the increase of *U2af1* expression by mTOR as shown in Figure 1C, would also accompany an increase in *U2af1b* expression, although with a lower degree. Thus, our results suggest that upon mTOR activation, an additional active suppression mechanism(s) for exon 3b utilization and/or a promotion mechanism(s) for exon 3a inclusion exists. To test this idea, we performed an siRNA-mediated screen targeting various splicing regulators to identify potential mTOR-regulated factors that regulate *U2af1* isoform expression. After following the screening strategy presented in Figure 1D, which takes into account the effects of knockdown of these RBPs on the splicing of *U2af1* tandem exons and the expression profile changes of these RBPs in response to mTOR activation, *Srsf3, Srsf5*, and *Cpsf5* emerged as candidates of mTOR-regulated factors for *U2af1* isoform regulation (Figure 1E and Supplementary Figure S1A–B, F–J). *Srsf3* was of particular interest because, as opposed to *Srsf5* and *Cpsf5*, it not only passed our screening criteria (Figure 1D) but also displayed 3′-UTR shortening by APA, a recently characterized post-transcriptional signature in the mTOR-activated transcriptome (Supplementary Figure S1K–M) (19). The *Srsf3* knockdown significantly increased the expression of *U2af1b* about ∼3-fold while *U2af1a* transcript level was relatively unaffected, suggesting that SRSF3 has a suppressive role on the inclusion of exon 3b (Figure 1E and Supplementary Figure S1F). Consistent with our previous findings on the role of 3′-UTR shortening in the promotion of protein synthesis (18,19), polysome profiling of *Srsf3* transcripts and western blot analysis showed that the SRSF3 protein level significantly increased due to the 3′-UTR APA in *Tsc1*^−/−^ compared to WT MEFs. (Figure 1F and Supplementary Figure S1N and O). A further analysis of *U2af1* transcript variants showed that the knockdown of *Srsf3* coupled with the inhibition of nonsense-mediated mRNA decay by puromycin treatment increased the expression of *U2af1c* transcript (not annotated in mouse mm10, the same structure of the transcript is annotated as NM_001025204 in human hg38) which contains both exons 3a and 3b (Figure 1G) (8,24,25). These results suggest that the exon 3b inclusion is actively suppressed, while the exon 3a selection is constitutively active. We next made a reporter construct containing a genomic DNA fragment of *U2af1* gene ranging from exon 2 to exon 4 (Supplementary Figure S1P). We then manipulated the cellular level of SRSF3 in *Tsc1^−/−^* MEFs harboring the reporter construct by transient overexpression and used qPCR to measure the selection of *U2af1* tandem exons from the reporter construct. Consistent with the measurements for endogenous *U2af1* isoform expression, the overexpression of SRSF3 in the *Tsc1^−/−^* MEFs significantly reduced the inclusion of exon 3b from the reporter construct (Supplementary Figure S1Q). Together, these results identify SRSF3 as one of the factors that contributes to *U2af1* tandem exon splicing by mTOR signaling and establish a regulatory pathway of U2AF1 isoform expression: a transcriptional activation of *U2af1* gene upon mTOR activation constitutively selects exon 3a for its splicing and inclusion while suppressing the inclusion of exon 3b, thus driving the biased expression of U2AF1a (Figure 1H).

### U2AF1 isoform-specific transcriptomes display an overlapping but distinctive alternative splicing profile

**Figure 2.**
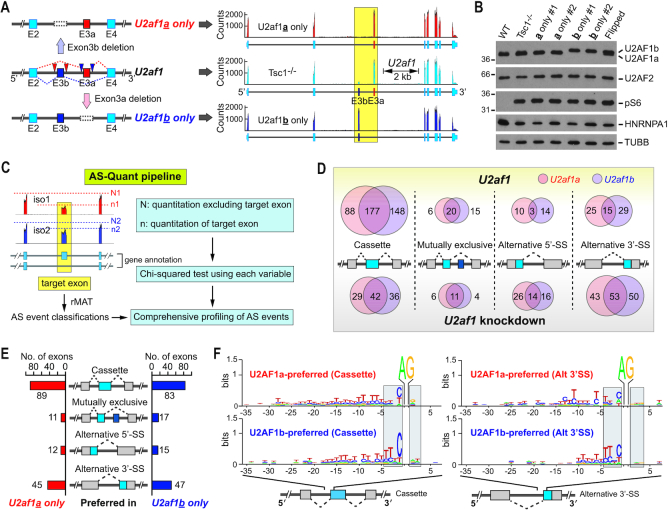
U2AF1 isoforms display distinctive alternative splicing profiles. (**A**) (left) Schematic for the generation of U2AF1 isoform-specific cell lines in *Tsc1^−/−^* MEFs. Location of guide RNA (gRNA) pairs to produce U2af1**a**-only and U2af1**b**-only cells are indicated by red and blue triangles, respectively. (right) RNA-Seq read alignments of *U2af1* gene locus in U2af1**a**-only, U2af1**b**-only and control *Tsc1^−/−^* MEFs. The yellow box highlights tandem exon regions in *U2af1*. (**B**) Western blot analyses of U2af1**a**-only, U2af1**b**-only, control *Tsc1^−/−^*, and WT MEFs. Exon 3a targeting experiment created several heterozygous clones, which were named as ‘flipped’ since the U2AF1a/U2AF1b ratio is flipped compared to control *Tsc1^−/−^* MEFs. A flipped clone is also loaded to aid visualizing the migration shift of U2AF1 isoforms. Two **a**-only and **b**-only cell lines were analyzed. (**C**) Schematic of custom-developed AS-Quant (*A*lternative *S*plicing *Quant*itation) pipeline for a quantitative analysis of alternative splicing. (**D**) Types of alternative splicing events dependent on the cellular level of U2AF1a or U2AF1b isoform. *U2af1* knockdown-dependent alternative splicing events are categorized and the numbers of events identified in U2af1**a**-only cell line (orange circles), U2af1**b**-only cell line (purple circles), and in both cell lines (overlapped regions) are presented. (upper) Number of alternative exons that are more included in the presence of *U2af1*. (lower) Number of alternative exons that are more included in the absence of *U2af1* (upon knockdown). (**E**) Types of alternative splicing events preferred by U2AF1a or U2AF1b. Alternative splicing events identified by a direct comparison between U2af1**a**- and U2af1**b**-only cell lines are presented. Alternative splicing events are categorized and the number of exons that are preferentially included in U2af1**a**-only (left) and U2af1**b**-only cell line (right) are shown. (**F**) The frequency of upstream nucleotides of the 3′-splice site of cassette type (left) or alternative 3′-splice site type (right) preferred by U2AF1 isoforms. The certainty (bit = log(frequency/2.4), ranging from 0 to 1.5) of nucleotides in each position of the upstream intron and the downstream exon regions from the AG dinucleotide of the 3′-splice site is illustrated. X-axis denotes the position of upstream and downstream nucleotides from the AG dinucleotide and Y-axis represents the certainty of the nucleotides.

Although two isoforms are produced from *U2AF1*, most studies on U2AF1 and its mutations do not distinguish functional differences that might be conferred by these isoforms (6,14,26–36). However, our above findings indicate that the stoichiometry of U2AF1 isoforms may change depending on cellular contexts. In fact, western blot analyses of U2AF1 showed that the stoichiometry of U2AF1 isoforms is dynamic across the tested cell lines, confirming that the cellular contents of U2AF1 isoforms are diverse and further suggesting that U2AF1a isoform cannot be presumed to be predominantly expressed in every biological or cellular model (Supplementary Figure S2A). To comprehensively understand how U2AF1 isoforms differentially contribute to the transcriptome, we adopted the CRISPR/Cas9 genome editing tool to create cell lines that only express one of the two U2AF1 isoforms in *Tsc1*^−/−^ MEF background. To separately knockout each of the *U2af1* isoforms, we designed pairs of CRISPR/Cas9 constructs that could create double-stranded breaks flanking one of the tandem exons to induce non-homologous end joining (NHEJ), leading to the removal of the targeted exon. The resulting *U2af1* locus would only have one usable exon 3, exon 3a or exon 3b, achieving the creation of U2af1**a**- or U2af1**b**-only cell lines (Figure 2A (left panel) and Supplementary Figure S2B). The exclusive expression of one isoform is confirmed by RNA-Seq and western blot analyses (Figure 2A (right panel) and B). We selected two clones of U2af1**a**- and U2af1**b**-only *Tsc1*^−/−^ MEF cell lines for future experiments to avoid artifacts from clonal variations. Of note, CRISPR/Cas9 genome editing did not drastically alter the overall expression of U2AF proteins, nor affect the cellular mTORC1 activity, as evidenced by western blot analyses on U2AF1, U2AF2 and phospho-S6 (Figure 2A and B).

To examine the transcriptome-wide changes of gene expression by U2AF1 isoforms, we performed RNA-Seq experiments using the #1 clone of U2af1**a**- and U2af1**b**-only cell lines in the presence or absence of *U2af1* knockdown. The knockdown of *U2af1* in these cell lines did not affect the level of U2AF2 (Supplementary Figure S2C). The analyses of RNA-Seq data from corresponding cell lines were focused on alternative splicing events using our custom-developed AS-Quant (*A*lternative *S*plicing *Quant*itation; Figure 2C) pipeline. AS-Quant first applies rMATS (20) to categorize potential alternative splicing events into four categories (cassette type or skipped exon, mutually exclusive exons, alternative 5′-splice site, and alternative 3′-splice site) based on the mm10 UCSC mouse genome annotation. Then the quantitation of affected exons in a transcript compared to the rest of exons in the transcript is further tested by the Chi-squared method to determine the alternative inclusion/exclusion of the tested exon between the two cases (Figure 2C).

Since a previous report showed that U2AF1a has a much broader impact on alternative splicing than U2AF1b (9), we asked whether U2AF1 isoforms have different capacities in alternative splicing. To this end, alternative splicing events in the RNA-Seq data of the control versus *U2af1* knockdown in U2af1**a**- or U2af1**b**-only cell line were analyzed using AS-Quant. Overall, we identified 568 exons in 451 genes to be U2AF1a-dependent alternative splicing events in U2af1**a**-only cell line, and 647 exons in 501 genes as U2AF1b-dependent alternative splicing events in U2af1**b**-only cell line; out of a total of 880 identified alternative splicing events, 335 of these exons are common in the two datasets (Figure 2D). Notably, cassette and mutually exclusive type of alternative splicing showed a much higher overlap than alternative 5′-splice site and 3′-splice site events between the two isoforms (Figure 2D). Moreover, in both knockdown experiments, the presence of U2AF1 is crucial for the exon inclusion in cassette type alternative splicing (upper far left of Figure 2D) and alternative uses of 3′-splice sites (lower far right of Figure 2D), supporting the suggested role of U2AF1 in exon inclusion/definition and 3′-splice site definition upon splicing (Supplementary Tables S1 and S2) (31,37,38). Collectively, these results show that the two U2AF1 isoforms have similar capacities to function as alternative splicing regulators but they appear to have different specificities.

Therefore, to further examine the functional differences of U2AF1 isoforms in alternative splicing regulation, we then sought to identify alternative splicing events that are differentially regulated by U2AF1 isoforms by directly comparing the RNA-Seq data from U2af1**a**- and U2af1**b**-only cell lines with AS-Quant. In this case, the data from cells expressing comparable levels of each U2AF1 isoform are directly compared, without considering the data from knockdown experiments. This approach excludes alternative exons that are redundantly regulated by U2AF1 isoforms or other RBPs and only reveals exons that are differentially regulated by the splicing machineries only harboring U2AF1a or U2AF1b. In this analysis, we identified 157 exons in 139 genes and 162 exons in 142 genes that are preferentially included in U2af1**a**- or U2af1**b**-only cells, respectively (Figure 2E and Supplementary Table S3). That is, 157 exons are more preferentially included and 162 exons are more preferentially skipped in an U2af1**a**-only environment compared to an U2af1**b**-only environment. To validate the alternative splicing events identified by AS-Quant, we randomly selected alternative splicing events and quantified these splicing events by RT-PCR using total RNAs purified from the two clones of U2af1**a**- and U2af1**b**-only cells (Supplementary Figure S2D). All tested alternative splicing events showed splicing patterns consistent with the RNA-Seq data analyses using AS-Quant. In addition, all tested genes showed similar splicing pattern changes within the U2af1**a**- or U2af1**b**-only clones, demonstrating that these alternative splicing events indeed show U2AF1 isoform preferences and did not arise from clonal variations (Supplementary Figure S2D).

Although U2AF1 has been indicated to bind to the consensus AG dinucleotide motif in the 3′-splice site, our analyses of U2AF1 isoform-dependent alternative splicing events strongly suggest that each U2AF1 isoform prefers additional distinct sequence contexts around the 3′-splice site and renders the specificity in exon choice for the splicing reaction. Therefore, we analyzed the nucleotide frequency surrounding the 3′-splice site of the cassette and alternative 3′-splice site type splicing events based upon U2AF1 expression as well as U2AF1 isoform preferences (–35 to +5 bp relative to the AG dinucleotide of 3′-splice site). In line with the well-established role of U2AF complex in splicing, exons whose inclusions are commonly promoted by U2AF1a and U2AF1b have a strong poly-pyrimidine tract frequency downstream of the –20 position compared to exons not promoted by U2AF1 (Supplementary Figure S2E and F). And interestingly, while sharing the feature of prominent poly-pyrimidine tract, nucleotide frequencies upstream of the 3′-splice site of U2AF1a-preferred exons have different sequence signatures compared to that of U2AF1b-preferred exons. Specifically, for both cassette and alternative 3′-splice site types, there is a higher frequency of C at the –1 position for U2AF1b-preferred exons. U2AF1b-preferred exons also have a stronger T preference at –3 and –4 positions (Figure 2F and Supplementary Figure S2E). In contrast, U2AF1a is less selective in the sequence preference at these positions and shows a weaker preference to polypyrimidine tracts compared to U2AF1b (Figure 2F and Supplementary Figure S2E). These analyses indicate that the two types of U2AF dimers consisting of two different U2AF1 isoforms have distinct nucleotide-binding preferences at the splice site. Together, these data provide evidence that the two isoforms have comparable involvements in the general splicing mechanism, yet a subset of alternative exons are differentially regulated by the two isoforms, demonstrating the functional differences between U2AF1a and U2AF1b in alternative splicing.

### U2AF1 isoform stoichiometry is a mechanistic factor for mutually exclusive alternative splicing

As shown above and in other studies (13,39–42), genes with tandem duplicated exons can produce highly similar isoforms with distinct functions. Therefore, it is important to understand how the mutually exclusive tandem duplicated exons are processed, which can involve more dynamic reorganization of RBPs and *cis*-acting sequence elements in introns and exons compared to other types of alternative splicing. Intriguingly, AS-Quant identified a number of tandem duplicated mutually exclusive alternative splicing events to be differentially regulated by U2AF1 isoforms. Although previous reports suggest that U2AF1 functions in alternative splicing of several mutually exclusive duplicated tandem exons, mechanistic insights of these alternative splicing events are largely lacking (23,28). Moreover, these studies did not consider the relevance of the functional differences of U2AF1 isoforms in the regulation of mutually exclusive alternative splicing events.

**Figure 3.**
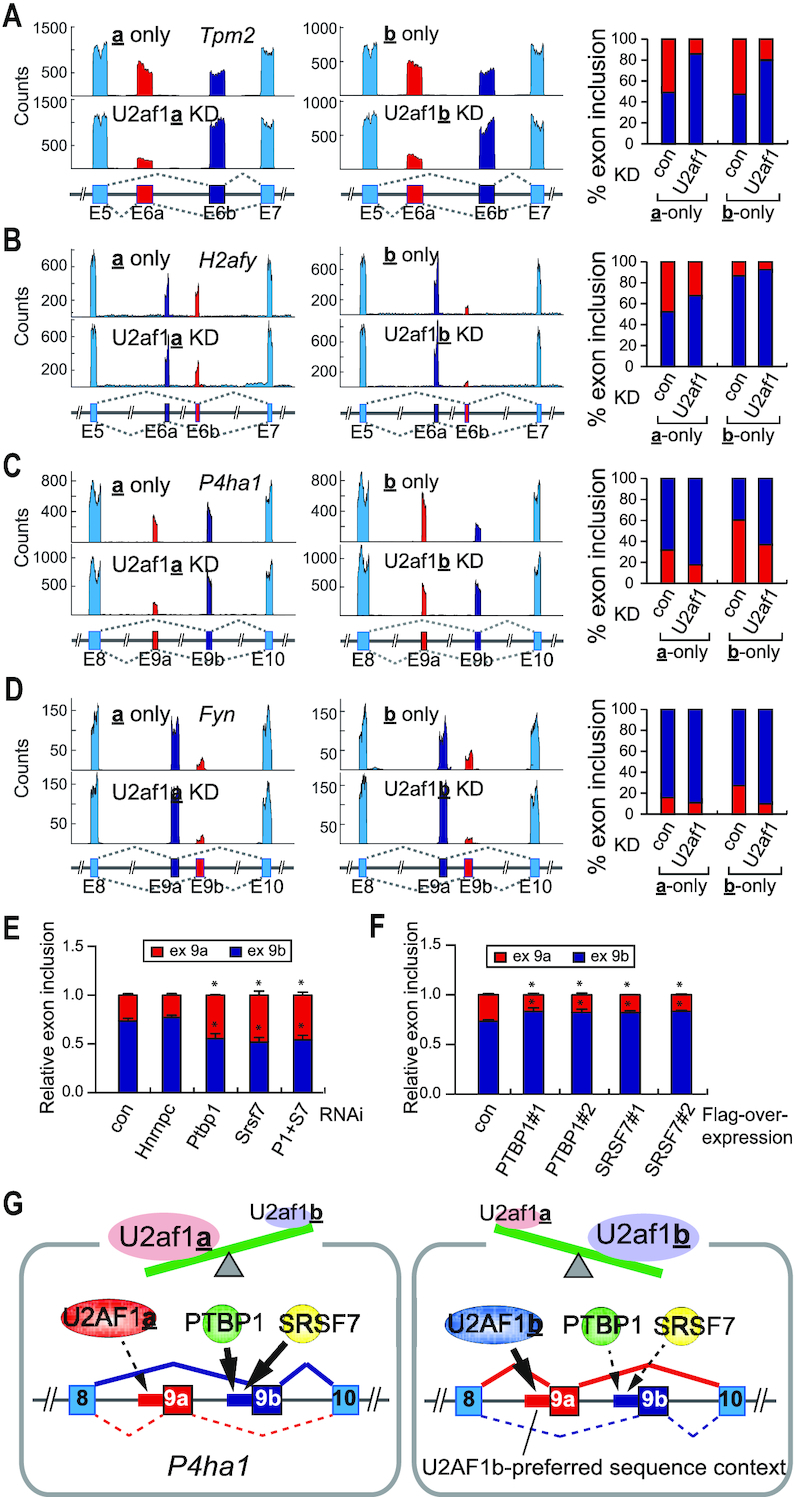
Stoichiometry of U2AF1 isoforms determines alternative 3′-splice site. (**A–D**) RNA-Seq read alignments of *Tpm2* (A), *H2afy* (B), *P4ha1* (C) and *Fyn* (D) gene loci in U2af1**a**-only, U2af1**b**-only and *U2af1* knockdown in corresponding cells. Please note that the designation of mutually exclusive exon a and b of these genes is in the order of exons from 5′ to 3′-end direction for convenience. Inclusion of exon a or b is shown based on the quantitation of RNA-Seq data with the matching color code. (**E**) An RNAi screening to identify a factor(s) for *P4ha1* exon 9b alternative splicing. Relative inclusion of exon 9a or exon 9b in *P4ha1* expression was presented after the knockdown of indicated RNA-binding proteins (RBPs) in *Tsc1*^−/−^ MEFs. The asterisks indicate the statistically significant decrease in the inclusion of exon 9b compared to the control. The data are presented as the mean (SD) (*P <0.0062, two-tailed Student's *t* test, *n* = 3 for technical repeats). (**F**) The effect of PTBP1 and SRSF7 overexpression on the inclusion of *P4ha1* exon 9b. PTBP1 or SRSF7 was overexpressed in *Tsc1*^−/−^ MEFs and the relative inclusion of exon 9b was measured. Two independent repeats of the experiments are shown. The asterisks indicate the statistically significant increase in the inclusion of exon 9b compared to control. The data are presented as the mean (SD) (*P <0.01, two-tailed Student's *t* test, *n* = 3 for technical repeats). (**G**) A proposed model for the mutually exclusive alternative splicing of *P4ha1* upon the changes of U2AF1 isoform stoichiometry. The stoichiometry of U2AF1 isoforms in cells determines the usage of one of the tandem exons’ splice site based on the nucleotide composition and the other splice site is selected by other splicing factors. In this case, PTBP1 and SRSF7 are one of the splicing factors involved in the mutually exclusive alternative splicing of *P4ha1*.

In our datasets, RNA-Seq read alignments of several duplicated tandem exons displayed mutually exclusive alternative splicing when comparing U2af1**a**- and U2af1**b**-only cells; moreover, RNAi knockdown of *U2af1* in those cells also showed characteristic U2AF1 isoform-dependent changes of mutually exclusive alternative splicing. For example, the inclusion of exon 6a in the mutually exclusive alternative splicing of *Tpm2* is dependent upon the overall level of U2AF1, but independent of which U2AF1 isoforms is present, as the shift of exon inclusion to 6b occurred similarly in the RNAi knockdown of *U2af1* in both U2af1**a**- and U2af1**b**-only cells (Figure 3A). In contrast, U2AF1-isoform dependent tandem exon splicing became apparent in the *H2afy* expression. In this case, the inclusion of exon 6b decreased as *U2af1* was knocked down in both isoform-specific cell lines. Notably, however, the inclusion of exon 6b was more favored in U2af1**a**-only cells compared to U2af1**b**-only cells (Figure 3B). These observations suggest that U2AF1a, as compared to U2AF1b, is more specific to exon 6b inclusion and is more capable of competing against exon 6a inclusion by unknown splicing factor(s). In the cases of *P4ha1* and *Fyn* expression, U2AF1b was more critical for the inclusion of a specific exon between the tandem exons. In *P4ha1* mutually exclusive alternative splicing, U2af1**b**-only cells exhibit more favorable exon 9a inclusion (59.9%) than U2af1**a**-only cells (31.0%) (Figure 3C). Furthermore, the knockdown of *U2af1* shifted the exon inclusion to 9b in both cell lines (40.1–63.5% in U2af1**b**-only cells and 69.0–83.5% in U2af1**a**-only cells) (Figure 3C). Similar observations were made in the expression of *Fyn*. In this case, exon 9b is more preferentially selected in U2af1**b**-only compared to U2af1**a**-only cells (25.7% versus 13.9% exon 9b inclusion) and the knockdown of *U2af1* in both cell lines decreased exon 9b inclusion (8.2% in **b**-only versus 9.5% in **a**-only). Thus, with a varying degree, it seems that U2AF1b is more specific than U2AF1a is to the splicing of *P4ha1* exon 9a and *Fyn* exon 9b; the other exon in those tandem exons is likely to be spliced by an unknown splicing factor(s) as both U2AF1 isoform knockdown increases the inclusion of the other exon (Figure 3C and D). Collectively, these results show that the stoichiometry of U2AF1 isoforms determines the selection of one of tandem exons for mutually exclusive alternative splicing and the other exon selection is completed by an unknown splicing factor(s). Indeed, a series of knockdown experiments on selected RBPs identified SRSF7 and PTBP1 as two of the splicing factors for *P4ha1* tandem exon splicing because the knockdown of *Srsf7* or *Ptbp1* in *Tsc1*^−/−^ MEFs decreased the inclusion of exon 9b (Figure 3E and Supplementary Figure S3A–C). Consistent with these observations, overexpression of *Ptbp1* or *Srsf7* in *Tsc1*^−/−^ MEFs increased the inclusion of exon 9b (Figure 3F and Supplementary Figure S3D). Together, these results support a model in *P4ha1* tandem exon splicing where U2AF1b has a higher preference to exon 9a inclusion than U2AF1a and furthermore, that PTBP1/SRSF7 have a role in the inclusion of exon 9b (Figure 3G). Therefore, the stoichiometry of U2AF1 isoforms and the level of competing splicing factors in a given cellular context likely determine the selection of tandem exons in mutually exclusive splicing. Of note, the concept that the stoichiometry of U2AF1 isoforms affects alternative splicing also applies to a simpler splicing type, e.g. cassette type (Supplementary Figure S3E–H). These demonstrate the importance of considering the functional differences of U2AF1 isoforms in mechanistic studies of the regulation of alternative splicing.

### Isoform-specific interactomes of U2AF1 feature common but refined cellular functions

**Figure 4.**
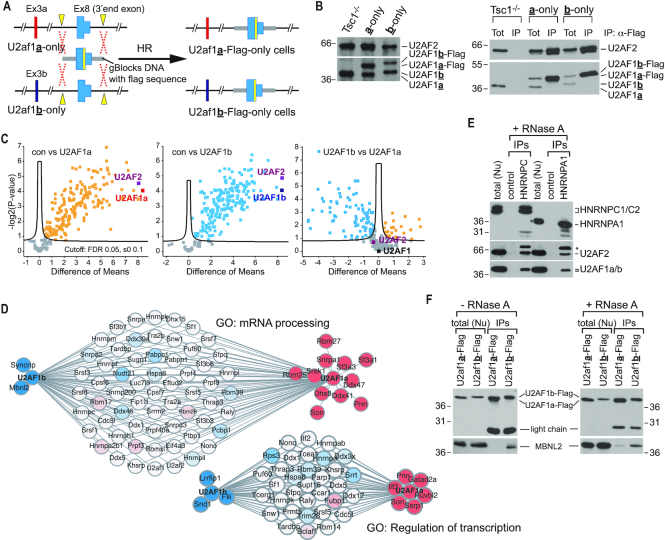
Overlapping but distinct interactome profiles of U2AF1 isoforms represents refined functional differences. (**A**) Schematic for CRISPR-induced homologous recombination (HR) to generate C-terminal Flag-tagged U2AF1 isoform-specific cell lines. Yellow rectangular box represents the Flag-tag. (**B**) Western blot analyses confirming the addition of a Flag-tag to U2AF1 (left). Anti-Flag immunoprecipitation (IP) and western blot analyses using total cellular extracts from Flag-tagged U2AF1 isoform-specific cell lines. Only Flag-tagged U2AF1 along with U2AF2 was immunoprecipitated (right). Tot: Total cell lysate, 1% of input was loaded. (**C**) Volcano plots illustrating enrichment of both U2AF1 isoforms and corresponding interactors. The plot compares the log2 mean protein LFQ intensity difference between the control, U2AF1a and U2AF1b baits against the logarthmic *P*-values. (**D**) Interactome analyses of U2AF1a and U2AF1b. Interactomes of U2AF1a and U2AF1b in GO term mRNA processing (GO:0006397) and regulation of transcription (GO:0006355) are illustrated. Proteins colored in solid blue and red represent unique interactors of U2AF1b and U2AF1a, respectively. (**E, F**) Co-IP and western blotting validation of U2AF1 isoform interactome analysis. (E) Anti-HNRNPC1/C2 or HNRNPA1 antibodies were used for co-IPs in the presence of RNase A. Nuclear fraction of HEK293 cells was used for co-IPs. 2.5% of input was loaded as total. The asterisk denotes a non-specific band which may come from undissociated antibody chains. (F) MBNL2 and U2AF1a-Flag or U2AF1b-Flag were co-expressed in HEK293 cells. Flag-IP was performed with nuclear fractions in the absence or presence of RNase A. 10% of input was loaded as total. Anti-Flag and Anti-MBNL2 antibodies were used for immunoblotting.

Differences in nucleotide preference and splicing regulation by U2AF1 isoforms raise the question of whether they form different functional complexes in cells. To identify proteins interacting with U2AF1 isoforms, we performed CRISPR-induced homologous recombination to insert a C-terminal Flag-tag to *U2af1* gene in U2af1**a**-only and U2af1**b**-only cell lines (Figure 4A and Supplementary Figure S4A). Characterization of the resulting Flag-tagged U2AF1 isoform-specific cell lines by western blots indicated that the Flag-tag was added to one allele of the *U2af1* gene in both U2af1**a**-only and U2af1**b**-only cell lines (Figure 4B, left). Immunoprecipitation (IP) with an α-Flag antibody followed by western blots confirmed that endogenous Flag-tagged U2AF1 isoforms pull down U2AF2, providing evidence that Flag-U2AF1 isoforms form endogenous U2AF complexes (Figure 4B, right). Since protein-protein interactions in the spliceosome are highly dynamic and transient, to capture the interactomes of U2AF1 isoforms, we performed the proteomics part of ribo-proteomics approach using formaldehyde-mediated crosslinking and Flag-IP in the presence of RNase A followed by mass spectrometry analysis (43,44). Mass spectrometry analysis of the immunoprecipitated samples showed high enrichment of U2AF heterodimer, suggesting the enrichment of U2AF1-interacting proteins in the co-IP (Figure 4C). We used the intensity based label-free quantification (LFQ) algorithm to assess the relative abundance of interactors normalized to each U2AF1 isoform (Supplementary Figure S4B). From this approach, we identified 127 U2AF1a interactors and 192 U2AF1b interactors significantly enriched over control Flag-IP (Figure 4C, Supplementary Figure S4C and Supplementary Table S4). Of these identified interactors, 23 and 88 proteins were specific to U2AF1a and U2AF1b, respectively (Figure 4C, Supplementary Figure S4C and Supplementary Table S4). Gene Ontology (GO) term analyses of the U2AF1 interactomes show that overall U2AF1a and U2AF1b interactomes are similar to each other and are highly enriched for the GO terms ‘mRNA processing’ and ‘splicing processes’ (Figure 4D and Supplementary Figure S4D and E). However, U2AF1a displays a greater association with interactors belonging to ‘mRNA processing’ and ‘splicing processes’ while U2AF1b interactors are highly enriched in ‘translation’ (Supplementary Figure S4D). Thus, our data suggest that the isoforms of U2AF1 form overlapping yet distinct protein complexes. Importantly, all enriched GO terms contain a subset of proteins exclusive to either U2AF1 isoform (Figure 4D and Supplementary Figure S4E). For instance, SF3A1 is specific to the U2AF1a interactome while MBNL2 is exclusive to the U2AF1b interactome (Figure 4D). To validate these results, we first conducted co-IP and western blot analyses using antibodies specific to identified interactors (HNRNPC1/C2 and HNRNPA1) of both U2AF1 isoforms in the presence of RNase A. As shown in Figure 4E, both HNRNPC1/C2 and HNRNPA1 directly interact with U2AF complexes endogenously. To further confirm the isoform-specific interactomes of U2AF1, we co-expressed MBNL2 (identified to bind to U2AF1b but not U2AF1a) and U2AF1a-Flag or U2AF1b-Flag in HEK293 cells and performed Flag-IP in the presence or absence of RNase A, followed by western blot analyses. As shown in Figure 4F, MBNL2 prefers to bind to U2AF1b over U2AF1a; this bias is not RNase dependent. Together, these validate the results of U2AF1 interactome analyses, which strongly suggest that U2AF1 isoforms have overlapping cellular functions yet provide refined or different regulatory roles by forming distinctive protein complexes.

### U2AF1a-mediated 5′-UTR alternative splicing promotes translation

**Figure 5.**
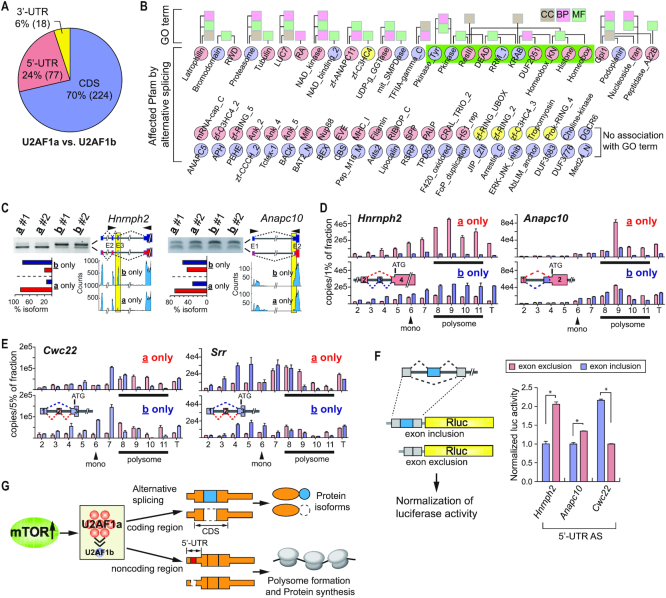
U2AF1 isoform-regulated alternative splicing in 5′-UTR modulates translation. (**A**) Distribution of alternative splicing in the regions of mRNA. Alternative splicing events displaying differences between U2af1**a**- and U2af1**b**-only cells were shown. (**B**) Affected Pfam domains by U2AF1 isoform-coordinated alternative splicing events were analyzed and their linkage to GO term is presented. Pfam domains affected by U2AF1a and U2AF1b-mediated alternative splicing are highlighted in light red and light blue, respectively. The Pfam domains highlighted in yellow are affected by both U2AF1a and U2AF1b-mediated alternative splicing. CC: Cellular Components; BP: Biological Processes; MF: Molecular Functions (**C**) Examples of 5′-UTR alternative splicing events in U2af1**a**- and **b**-only cells. RT-PCR and agarose gel electrophoresis were conducted to validate alternative splicing events. RNA-Seq read alignments and quantitation of alternative splicing events are shown. Arrows indicate the position of primer binding sites for RT-PCR analyses. Splicing isoforms and their quantitation are color-coded as illustrated; yellow boxes highlight the alternative exons. Asterisk denotes a non-specific PCR product. (**D**) Polysome profiling analyses on the cytosolic fraction of U2af1**a**-only and U2af1**b**-only cells. Distribution of 5′-UTR alternative splicing transcripts (left, *Hnrnph2*; right, *Anapc10*) in polysome fractionation were analyzed by absolute quantitation using qPCR. T: 10% of input. Splicing isoforms are color-coded as illustrated. Monosome and polysome fractions are indicated. (**E**) The same analyses described in (D) were conducted on *Cwc22* and *Srr* genes. (**F**) Luciferase assays showing the effects of 5′-UTR alternative splicing events on translation efficiency. The 5′-UTRs including or excluding the alternative exons of *Hnrnph2, Anapc10*, and *Cwc22* were placed into the 5′-UTR of luciferase reporter. The fold-changes of luciferase signals between the exon-included and exon-excluded 5′-UTR reporter construct pairs of the three genes are shown in bar graphs. The data are presented as the mean (SD) (*P < 8.6e-5, two-tailed Student's *t* test, *n* = 4 for technical repeats). (**G**) A proposed model for U2AF1 isoform-coordinated translational regulation by 5′-UTR alternative splicing and the connection to mTOR signaling. In this model, mTOR-regulated changes of U2AF1 expression profile contributes to the proteome regulation by multiple ways. Alternative splicing in coding regions produce protein isoforms while alternative splicing in the 5′-UTR regulates differential translation.

To understand the physiological consequences of U2AF1 isoform-mediated alternative splicing events (Figure 2E), we surveyed regions of these alternative splicing events and found that 70% of the alternative splicing events affect the coding capacity of genes while 24% and 6% of the alternative splicing events occur in the 5′-UTR and 3′-UTR, respectively (Figure 5A). This distribution is very similar to that of the known alternative splicing events in the mouse genome (mm10, Supplementary Figure S5O). To look into the functional proteomes regulated by U2AF1 isoforms, we first searched the alternative splicing events affecting coding DNA sequence (CDS) regions against Pfam domain database. Out of the 224 CDS alternative splicing events, 64 events affected 74 functional domains annotated by Pfam. Among those, 28 Pfam domains were associated with the GO term while 46 Pfam domains were not (Figure 5B). Almost a half of the GO term-associated Pfam domains including ‘Pkinase_Tyr’ and ‘Homeobox’ clustered together (green box in Figure 5B). Collectively, these indicate that the alternative splicing events differentially regulated by U2AF1 isoforms could widely affect various cellular pathways.

Interestingly, the average length of 5′-UTR of transcripts with annotated alternative splicing events is 527.7 nucleotides, which is much longer than that of 5′-UTR of transcripts without reported alternative splicing events (226.6 nucleotides) in the mouse transcriptome (Supplementary Figure S5A). In addition, the relative proportion of alternative exon length to the entire 5′-UTR is about 29.9% (Supplementary Figure S5B). Since 5′-UTR is known to contain diverse elements for translational regulation (45,46), it is presumed that the alternative splicing events in the 5′-UTR reconfigure these regulatory *cis*-elements. Indeed, a search for potential regulatory elements in the affected 5′-UTRs in our data (U2af1**a**-only versus U2af1**b**-only) using UTRScan (47) identified several known 5′-UTR motifs and upstream open reading frames (uORFs) that were reconfigured by U2AF1 isoform-regulated alternative splicing events (Supplementary Figure S5C and D). Out of the 77 genes showing U2AF1 isoform-mediated 5′-UTR alternative splicing events, 35 genes are predicted to reconfigure one or more uORFs (Supplementary Figure S5D). In this case, not only the frequency but also the average length of uORFs were significantly changed by alternative splicing in the 5′-UTR.

RNA-Seq read alignments and semi-quantitative analyses of several genes on 5′-UTR alternative splicing validated U2AF1 isoform-specific events in all tested U2af1**a**- and U2af1**b**-only cells (Figure 5C and Supplementary Figure S5E). To examine whether these 5′-UTR alternative splicing events are associated with translational regulation, we conducted polysome fractionation using the cytoplasmic extracts from U2af1**a**- and U2af1**b**-only cells (Supplementary Figure S5F) and analyzed the distribution of two alternative 5′-UTR isoforms by qPCR with absolute quantitation. *Hnrnph2* is mostly expressed as the exon 3-skipped isoform in U2af1**a**-only cells whereas the exon 3-included isoform is highly expressed in U2af1**b**-only cells (Figure 5C). Our quantitative analyses showed that, given the input amounts, the *Hnrnph2* exon 3-skipped isoform (shown in light red) formed polysomes more efficiently in both U2af1**a**- and U2af1**b**-only cells, while the transcript with exon inclusion (shown in light blue) was less efficient in forming polysomes (Figure 5D, left and Supplementary Figure S5G, M). Similar differential polysomal distributions due to alternative splicing were observed in *Anapc10* (Figure 5D right and Supplementary Figure S5H, M). Interestingly, exon skipping is not always favored for polysome formations. In the case of *Cwc22* and *Srr* where exon inclusion in the 5′-UTR occurs more often in U2af1**a**-only cells (Supplementary Figure S5I–J, M), the exon 2-included isoforms of both *Cwc22* and *Srr* (shown in light red) formed polysomes more efficiently compared to the exon 2-skipped isoforms (Figure 5E and Supplementary Figure S5K–M). Interestingly, in these select genes, alternative splicing events promoted by U2AF1a (skipping in *Hnrnph2* and *Anapc10*, inclusion in *cwc22* and *Srr*) leads to increase in polysome formation of the transcripts. To validate the findings of polysome fractionation analyses, we cloned the 5′-UTRs of *Hnrnph2, Anapc10, Cwc22*, and *Pex2*, including or excluding the alternative exon, into the 5′-UTR of luciferase reporter constructs (Figure 5F and Supplementary Figure S5N). We then transfected these constructs into *Tsc1^−/−^* MEFs and compared their luciferase activities to measure the effects of these 5′-UTRs on translation efficiency. As shown in Figure 5F and Supplementary Figure S5N, consistent with the results of polysome fractionation analyses, the exclusion of the 5′-UTR alternative exons of *Hnrnph2, Anapc10*, and *Pex2* and the inclusion of the 5′-UTR alternative exon of *Cwc22* lead to higher translation of luciferase compared to their counterparts. Together, these data show that the alternative splicing events in the 5′-UTR modulated by U2AF1 isoforms coordinate translation and suggest that the stoichiometry of U2AF1 isoforms plays a key role in the regulation of translation, potentially uncoupling the correlation between mRNA and protein abundance in cells.

## DISCUSSION

U2AF1 has been extensively studied for its crucial role in pre-mRNA splicing and the pathogenesis of myelodysplasia syndrome (MDS) (1,6,9,14,27–32,34–36,48–50). Albeit two isoforms are expressed from *U2AF1*, early studies on U2AF1 were not able to functionally differentiate two isoforms (8,9,49). Furthermore, commonly used mammalian cell lines often express more U2AF1a than U2AF1b (7–9). Accordingly, most, if not all studies on U2AF1 and its pathogenic mutations do not distinguish U2AF1 isoforms (6,9,14,26–35,48,51–53). A high sequence similarity along with the same molecular weight between the two isoforms make it challenging to study one isoform over the other. In addition, RNAi knockdown approaches for functional studies on U2AF1 isoforms are poised to generate U2AF1a-biased outcomes as many cell systems used for this kind of approach underrepresent U2AF1b expression (7–9). In contrast, our genome editing approach to produce both isoform-specific cell lines provides an unbiased biological system to understand the function of U2AF1 isoforms. In fact, unlike previous reports (7,9), our study could bring up underrepresented U2AF1b functions as our data show that the number of exons and genes exclusively regulated by each U2AF1 isoform is similar (Figure 2D and E). In conjunction with our interactome analyses, these indicate that two U2AF1 isoforms distinctively contribute to the transcriptome and have nuanced functional differences in cells. These conclusions are along the same lines with other well characterized tandem exon-derived isoforms including *PKM* and *FGFR2* (10–13,39). In this regard, our findings of distinctive functions of U2AF1 isoforms raise important questions regarding pathogenic U2AF1 mutations in MDS. Since most, if not all, studies on U2AF1 pathogenic mutations do not differentiate the two U2AF1 isoforms nor profile their expressions, they are not comprehensive in understanding the pathogenic mechanisms of MDS by not recognizing the potential functional impacts of the dynamic expressions of U2AF1 isoforms on the phenotypes that are attributed to U2AF1 mutations (26–28,34–36,50).

Obtaining the Flag-tagged endogenous U2AF1 isoform-specific cell lines with CRISPR-induced homologous recombination allowed the enrichment of U2AF1 isoform-specific interactomes by simply performing Flag-IP. More importantly, it eliminated the need to exogenously over-express bait proteins that may skew the stoichiometry of interactomes, and allowed us to IP endogenous U2AF1 isoform-specific complexes. The results of this comprehensive interactome analysis of U2AF1 isoforms suggest that the specificity of the isoform-specific interactomes could be a key characteristic of distinct functionality of the isoforms. Thus, these isoform-specific interactomes could not only help explain the different sequence preferences of the two isoforms, they could also represent previously unknown and/or sophisticated functions of U2AF1. Indeed, consistent with our findings of a possible link between U2AF1b and translation, a recent study suggested a role of U2AF1 and its mutations in the regulation of translation in the cytoplasm, although this study still lacked the information on U2AF1 isoforms (54). Collectively, these demonstrate that our approach of interactome analyses is beneficial in revealing unknown and sophisticated functions of U2AF1 isoforms.

One of the surprising outcomes of U2AF1 isoform-specific alternative splicing is the translational regulation through 5′-UTR alternative splicing. As shown by various computational analyses, alternative splicing in the 5′-UTR driven by U2AF1 isoform preferences as well as U2AF1 isoform knockdowns dynamically rearranges known *cis*-regulatory elements and uORFs (Supplementary Figure S5C–D, Q–S). Thus, 5′-UTR alternative splicing reprograms multiple features in the 5′-UTR and can regulate translation. One of the most well characterized molecular signatures of 5′-UTR in translational regulation is the translational activation of 5′-TOP (terminal oligopyrimidine) containing mRNAs by mTOR (55). Intriguingly, most 5′-UTR alternative splicing events specific to U2af1**a**- and U2af1**b**-only cells do not contain a 5′-TOP signature (only 2 out of 77 events in Figure 5A contain 5′-TOP feature, Supplementary Figure S5T). This pattern was consistent with the dataset of U2af1**a**-only control versus knockdown and U2af1**b**-only control versus knockdown (7 out of 130 events and 5 out of 132 events contain 5′-TOP feature, Supplementary Figure S5P, U). A recent study using transcription start site profiling reported that distinct classes of non-5′-TOP mRNAs were subjected to mTOR-regulated translational control (56). Interestingly, the study found that mTOR-dependent translation of these non-5′-TOP mRNAs have short or long 5′-UTRs and the length of 5′-UTR is associated with cellular pathways targeted by non-5′-TOP mRNAs (56). Our findings in this study present U2AF1 isoform-regulated alternative splicing in the 5′-UTR as a previously unrecognized translational regulatory mechanism and provide mTOR-regulated U2AF1 isoform profile as a molecular link between mTOR and non-5′-TOP mRNA translation (Figure 5G).

## DATA AVAILABILITY

The accession number for the RNA-Seq data in this study is SRP215854.

## Supplementary Material

gkz761_Supplemental_Files
