# Supplementary material for: mTOR-regulated U2af1 tandem exon splicing specifies transcriptome features for translational control
Source: Nucleic Acids Res. 2019 Sep 3;47(19):10373–87. doi: 10.1093/nar/gkz761 (PMC6821156; doi:10.1093/nar/gkz761)
Supplement: gkz761_Supplemental_Files [file gkz761_supplemental_files.zip › Supplementary Document_revised_Final2.pdf]

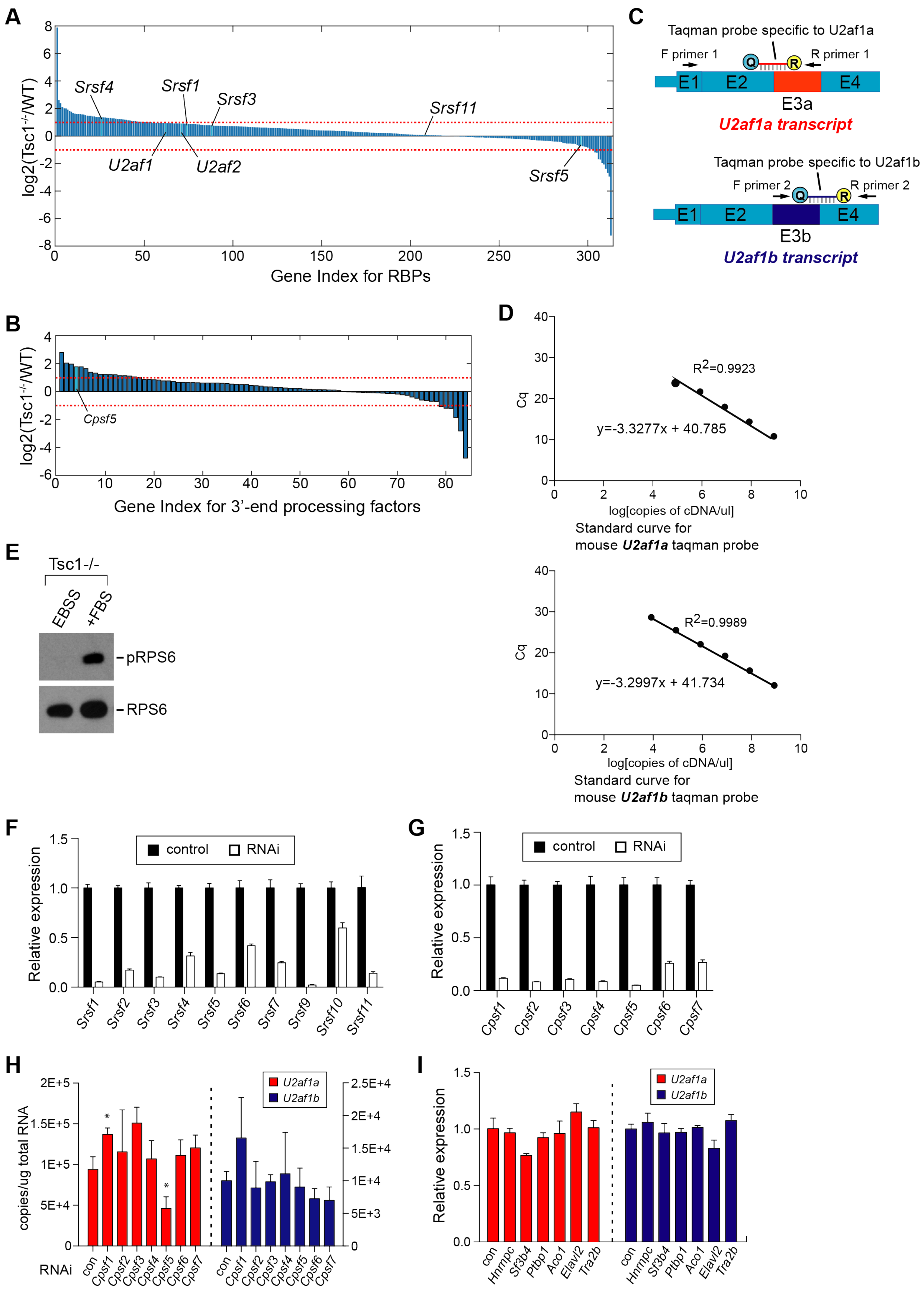
A
log2(Tsc1-/-/WT)
Gene Index for RBPs
Srsf4
Srsf1
Srsf3
Srsf11
U2af1
U2af2
Srsf5
B
log2(Tsc1-/-/WT)
Gene Index for 3'-end processing factors
Cpsf5
C
Taqman probe specific to U2af1a
F primer 1
R primer 1
E1
E2
E3a
E4
U2af1a transcript
Taqman probe specific to U2af1b
F primer 2
R primer 2
E3b
U2af1b transcript
D
Cq
R2=0.9923
y=-3.3277x + 40.785
log[copies of cDNA/ul]
Standard curve for
mouse U2af1a taqman probe
R2=0.9989
y=-3.2997x + 41.734
Standard curve for
mouse U2af1b taqman probe
E
Tsc1-/-
EBSS
+FBS
pRPS6
RPS6
F
Relative expression
control
RNAi
Srsf1
Srsf2
Srsf3
Srsf4
Srsf5
Srsf6
Srsf7
Srsf9
Srsf10
Srsf11
G
Cpsf1
Cpsf2
Cpsf3
Cpsf4
Cpsf5
Cpsf6
Cpsf7
H
copies/ug total RNA
U2af1a
U2af1b
RNAi
con
I
Hnrnpc
Sf3b4
Ptbp1
Aco1
Elavl2
Tra2b

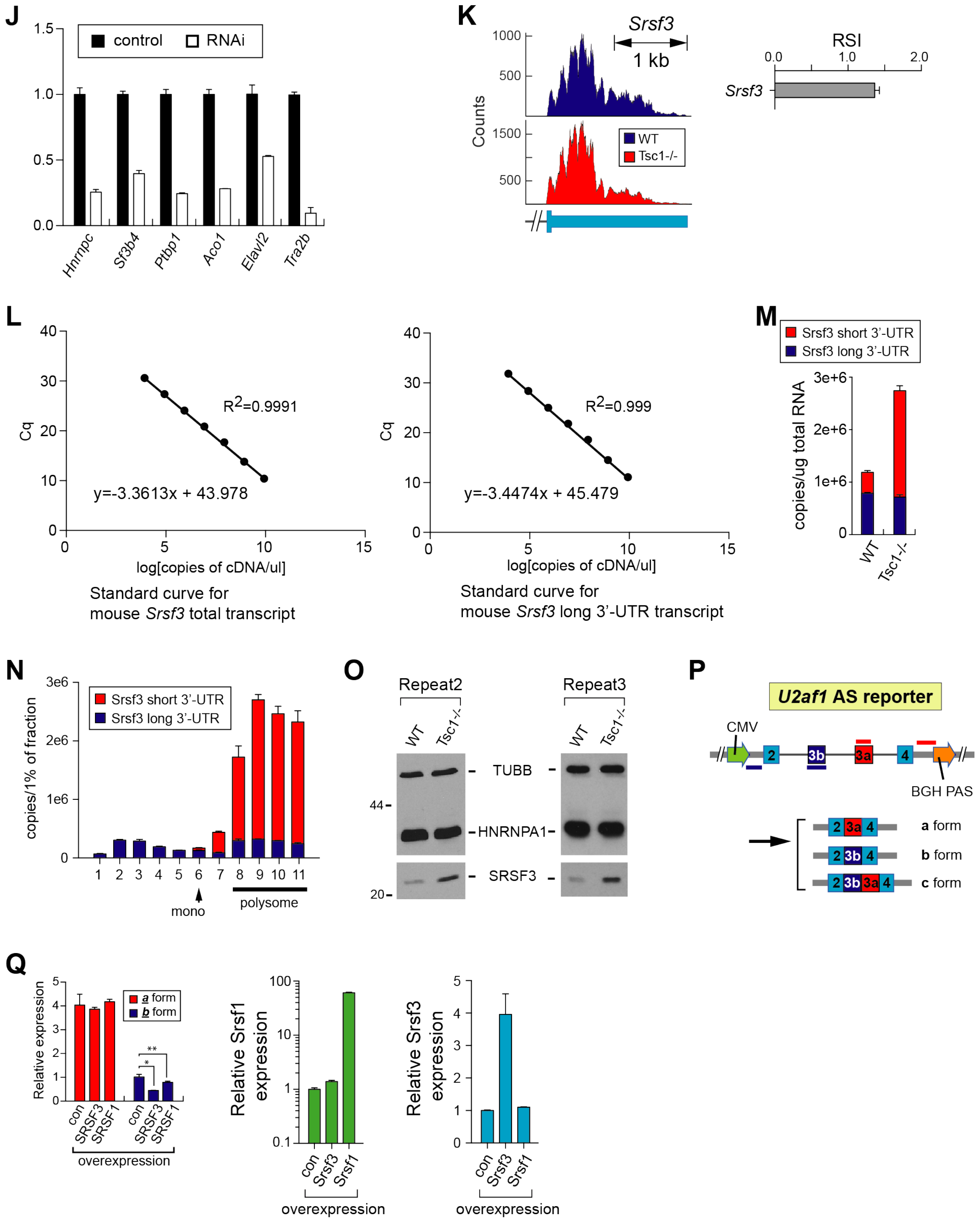
J
control
RNAi
1.5
1.0
0.5
0.0
Hnrnpc
Sf3b4
Ptbp1
Aco1
Elavl2
Tra2b
K
Srsf3
1 kb
1000
500
1500
500
Counts
WT
Tsc1-/-
RSI
0.0
1.0
2.0
Srsf3
L
40
30
20
10
0
Cq
R2=0.9991
y=-3.3613x + 43.978
0
5
10
15
log[copies of cDNA/ul]
Standard curve for
mouse Srsf3 total transcript
R2=0.999
y=-3.4474x + 45.479
log[copies of cDNA/ul]
Standard curve for
mouse Srsf3 long 3'-UTR transcript
M
Srsf3 short 3'-UTR
Srsf3 long 3'-UTR
3e+6
2e+6
1e+6
0
copies/ug total RNA
WT
Tsc1-/-
N
3e6
2e6
1e6
0
copies/1% of fraction
Srsf3 short 3'-UTR
Srsf3 long 3'-UTR
1 2 3 4 5 6 7 8 9 10 11
mono
polysome
O
Repeat2
Repeat3
WT
Tsc1-/-
TUBB
HNRNPA1
SRSF3
44
20
P
U2af1 AS reporter
CMV
2
3b
3a
4
BGH PAS
a form
b form
c form
Q
Relative expression
5
4
3
2
1
0
a form
b form
**
*
con
SRSF3
SRSF1
overexpression
Relative Srsf1 expression
100
10
1
0.1
con
Srsf3
Srsf1
overexpression
Relative Srsf3 expression
con
Srsf3
Srsf1
overexpression

Supplementary Figure 1. (**A, B**) Histogram plots of transcript expression level change in Tsc1$^{-/-}$ compared to WT MEFs of select RBPs (A) and 3'-end processing factors (B) based on RNA-Seq quantification. Genes mentioned in the main text are indicated. (**C**) A diagram showing the locations of Taqman probes and PCR primers used in Taqman qPCR assays on *U2af1* transcript isoforms. (**D**) Standard curves for absolute quantitation of mouse *U2af1* transcript isoforms using Taqman qPCR assay. (**E**) Western blot analysis on *Tsc1*$^{-/-}$ MEF cells under EBSS or FBS add-back treatment. The changes of mTOR activity by EBSS and FBS treatment are evidenced by pS6 (pRPS6) blotting, while S6 blotting serves as a loading control. (**F**) Knockdown of various SR splicing factors by RNAi. Relative expression of SR proteins in control and knockdown samples was measured using qPCR. (**G**) Knockdown of various polyadenylation factors by RNAi. Relative expression of CPSF proteins in control and knockdown samples was measured using qPCR. (**H**) Changes of *U2af1* isoform expression upon the knockdown of various polyadenylation factors. The amounts of *U2af1a* and *U2af1b* isoforms were measure by Taqman qPCR assay. (**I, J**) Small scale RNAi screen for regulators of alternative splicing. Each indicated splicing factor was knocked down by RNAi and the expression of *U2af1* isoforms was measured by Taqman qPCR assay. (**K**) *Srsf3* transcript undergoes 3'-UTR alternative polyadenylation (APA) upon the mTOR activation. (left) RNA-seq read alignments of the *Srsf3* 3'-UTR in WT and *Tsc1*$^{-/-}$ MEF cell lines. (right) RSI (relative shortening index) measurement of *Srsf3* 3'-UTR APA in *Tsc1*$^{-/-}$ relative to WT MEF cell lines. (**L**) Standard curves for absolute quantitation using qPCR assay on mouse *Srsf3 long 3'-UTR and total* transcripts. (**M**) Absolute quantitation of *Srsf3* long and short 3'-UTR transcripts using qPCR. The standard curves in (L) were used for the calculation. (**N**) Polysome profiling for *Srsf3* long and short 3'-UTR transcripts in *Tsc1*$^{-/-}$ MEFs. Absolute quantitation of long 3'-UTR and total *Srsf3* transcripts was conducted. The

amount of short 3'-UTR *Srsf3* transcript was calculated by the subtraction of long from total amounts. **(O)** The biological repeats of the Western blot analysis of SRSF3 in WT and *Tsc1*$^{-/-}$ MEFs shown in Fig. 1F. TUBULIN and HNRNPA1 were used as loading controls. (**P**) Schematic of *U2af1* tandem exon splicing reporter construct. (**Q**) Overexpression of SRSF3 suppresses exon 3b inclusion in *U2af1* splicing reporter assay. Two splicing isoforms from the reporter was measured by Taqman qPCR with relative quantitation upon the overexpression of SRSF3, SRSF1 and vector alone. Overexpression of *Srsf1* and *Srsf3* in the experiments was validated by qPCR with relative quantitation. The data are the mean (SD) (*p=0.010, **p=0.13 ; two-tailed Student's *t* test, n=3 for technical repeats).

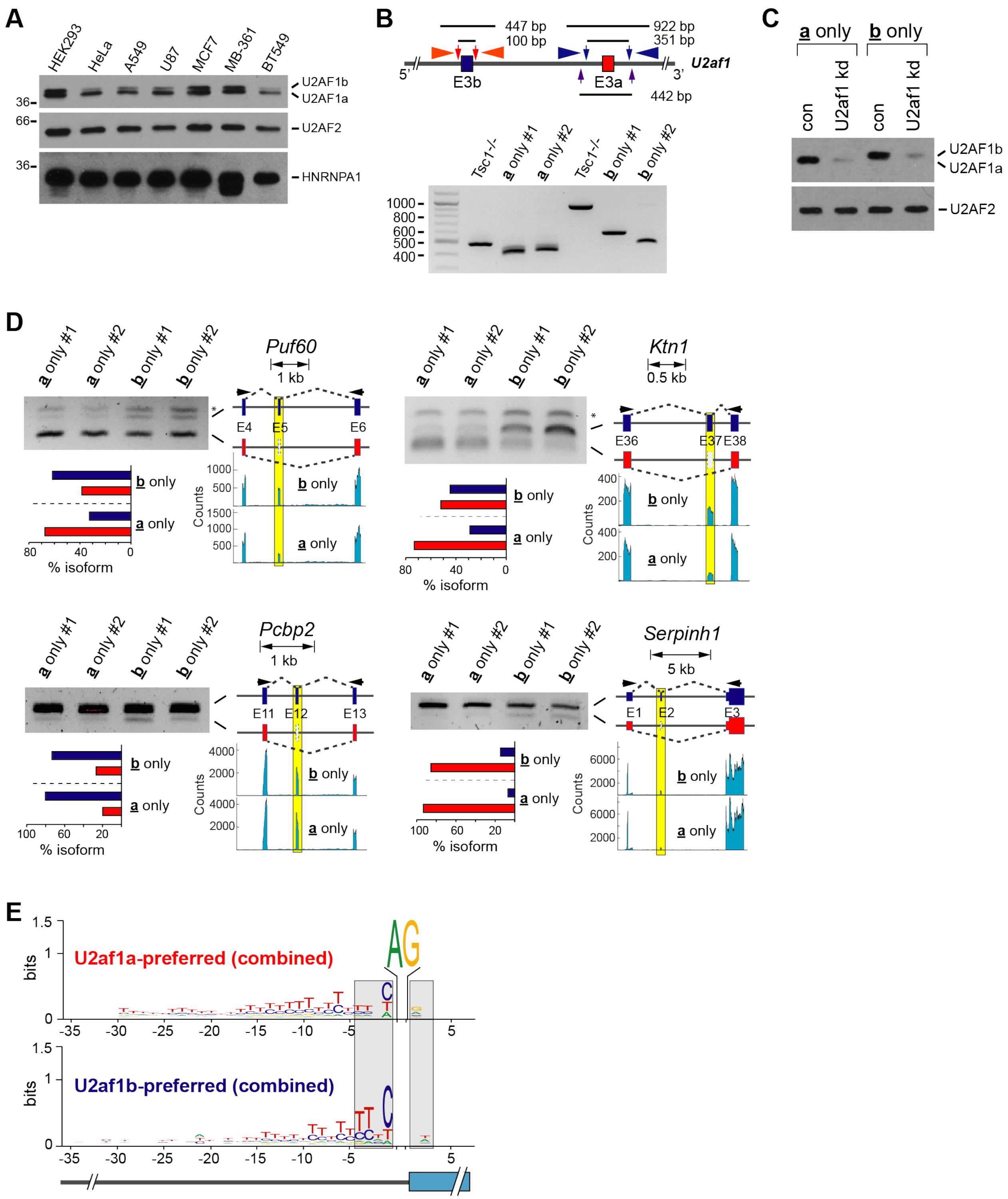
A
HEK293
HeLa
A549
U87
MCF7
MB-361
BT549
U2AF1b
U2AF1a
U2AF2
HNRNPA1
36
66
36
B
447 bp
100 bp
922 bp
351 bp
442 bp
E3b
E3a
U2af1
5'
3'
Tsc1-/-
a only #1
a only #2
Tsc1-/-
b only #1
b only #2
1000
800
600
500
400
C
a only
b only
con
U2af1 kd
con
U2af1 kd
U2AF1b
U2AF1a
U2AF2
D
Puf60
1 kb
E4
E5
E6
b only
a only
% isoform
Counts
Ktn1
0.5 kb
E36
E37
E38
Pcbp2
E11
E12
E13
Serpinh1
5 kb
E1
E2
E3
E
bits
U2af1a-preferred (combined)
U2af1b-preferred (combined)
-35
-30
-25
-20
-15
-10
-5
5

F

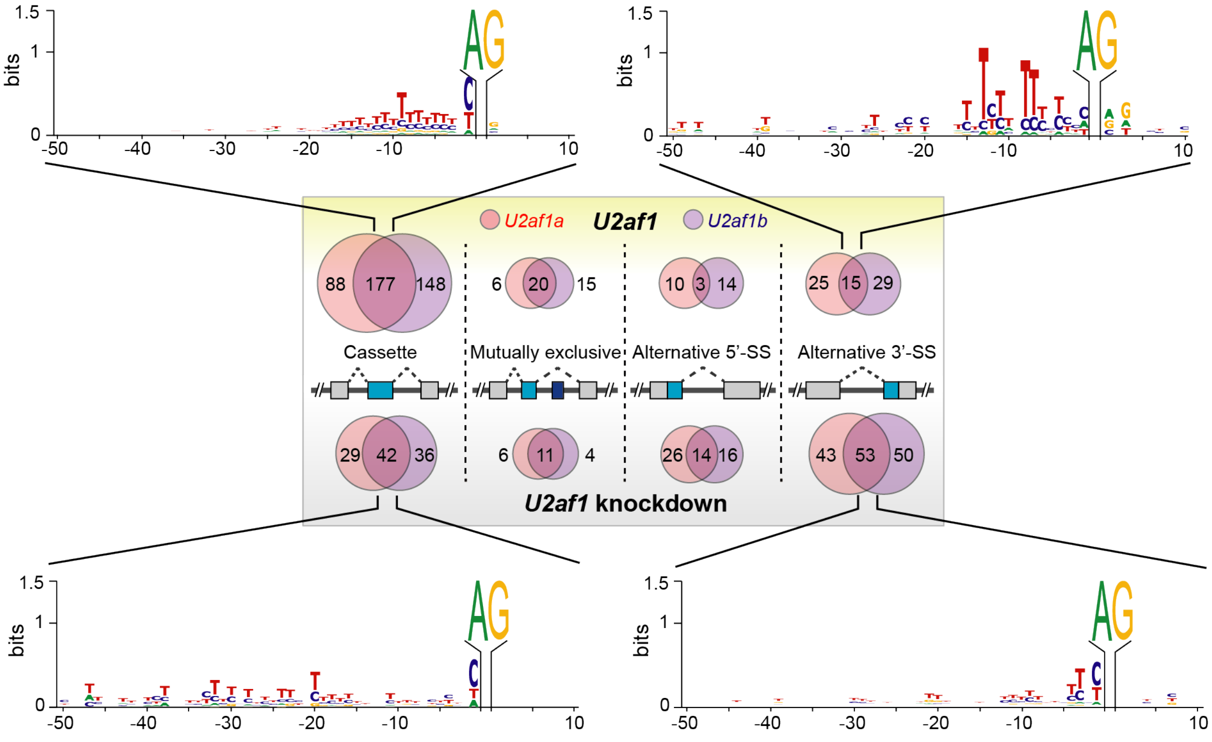

Supplementary Figure 2. (**A**) Western blot analyses of U2AF1 isoforms using various cancer cell lines. (**B**) (upper panel) A diagram illustrating the design of CRISPR/Cas9-mediated generation of U2af1**a**- and U2af1**b**-only cell lines. The locations of exon-flanking gRNAs are denoted by arrows. Primer sets used for gDNA screening are denoted by triangles. The distances between primer sets and the flanking gRNA pairs are indicated. (lower panel) PCR using the denoted gDNA screening primer sets followed by agarose gel electrophoresis. The downshift of the bands in U2af1**a**- and U2af1**b**-only cells demonstrates the successful homozygous editing by the exon-flanking gRNA pairs via NHEJ. (**C**) Western blot analysis on *Tsc1*$^{-/-}$ MEF, U2af1**a**-only#1, and U2af1**b**-only#1 cells treated with control (con) or U2af1-targeting siRNA (U2af1 kd). (**D**) Splicing of four representative alternative splicing events showing U2AF1 isoform preferences. For each panel, RNA-Seq read alignments and quantitation of alternative exon of U2af1**a**-only#1 and U2af1**b**-only#1 cell lines are shown. RT-PCR validation in U2af1**a**-only#1, U2af1**a**-only#2, U2af1**b**-only#1, and U2af1**b**-only#2 cell lines are shown. Splicing isoforms are

color-coded as illustrated; yellow boxes highlight the alternative exons. Asterisks denote non-specific bands. (**E**) The frequency of upstream nucleotides of 3'-splice site preferred by U2AF1 isoforms. The certainty of nucleotides in each position was calculated by considering both cassette and alternative 3'-splice site type splicing. (**F**) The frequency of upstream nucleotides of the 3'-splice site of exons commonly dependent on both U2AF1 isoforms. (upper) Exons that are more included in the presence of *U2af1*. (lower) Exons that are more included in the absence of *U2af1* (upon knockdown). (left) 3'-splice site of cassette type alternative splicing. (right) 3'-splice site of alternative 3'-splice site type alternative splicing.

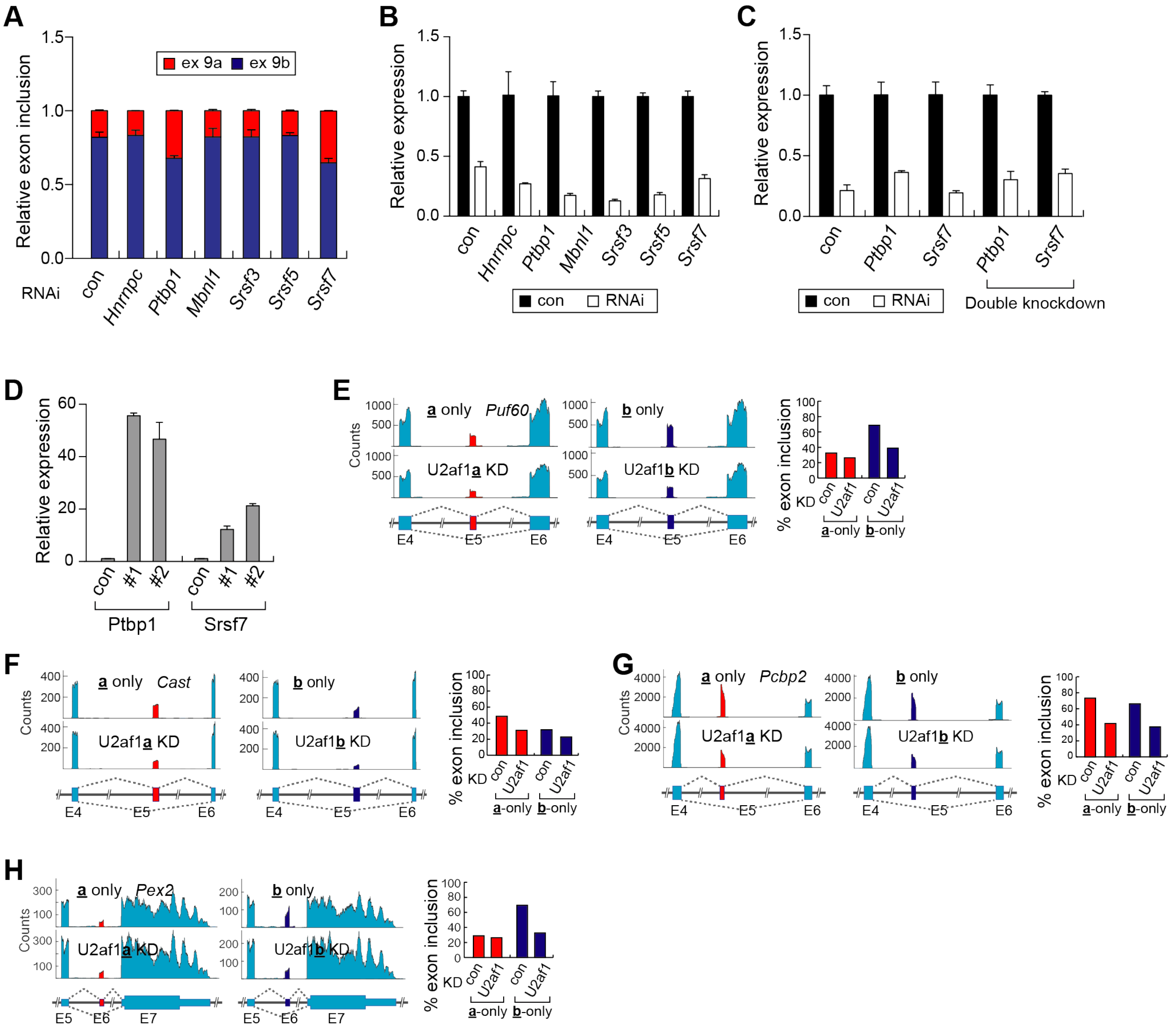

Supplementary Figure 3. (**A**) An initial RNAi screen for RBPs that promote the inclusion of exon 9b in *P4ha1*. Relative exon inclusion was calculated by relative quantitative PCR. (**B**) The measurement of knockdown efficiency of RBPs shown in (A). Relative qPCR was used for quantitation. (**C**) The measurement of knockdown efficiency of RBPs shown in Fig. 3E. Relative qPCR was used for quantitation. (**D**) The measurement of *Ptbp1* and *Srsf7* overexpression shown in Figure 3F. Relative qPCR was used for quantitation. **(E-H)** Examples of cassette type alternative splicing events that depend upon U2AF1 isoform in various manners. RNA-Seq read alignments of *Puf60* (preferring U2AF1b) (E), *Cast* (preferring U2AF1a) (F), *Pcbp2* (no U2AF1

isoform preference) (G) and *Pex2* (only dependent upon U2AF1b) (H) gene loci in U2af1**a**-only, U2af1**b**-only and *U2af1* knockdown in corresponding cells are shown. Inclusion of the alternative exon is plotted based on the quantitation of RNA-Seq data with the matching color code.

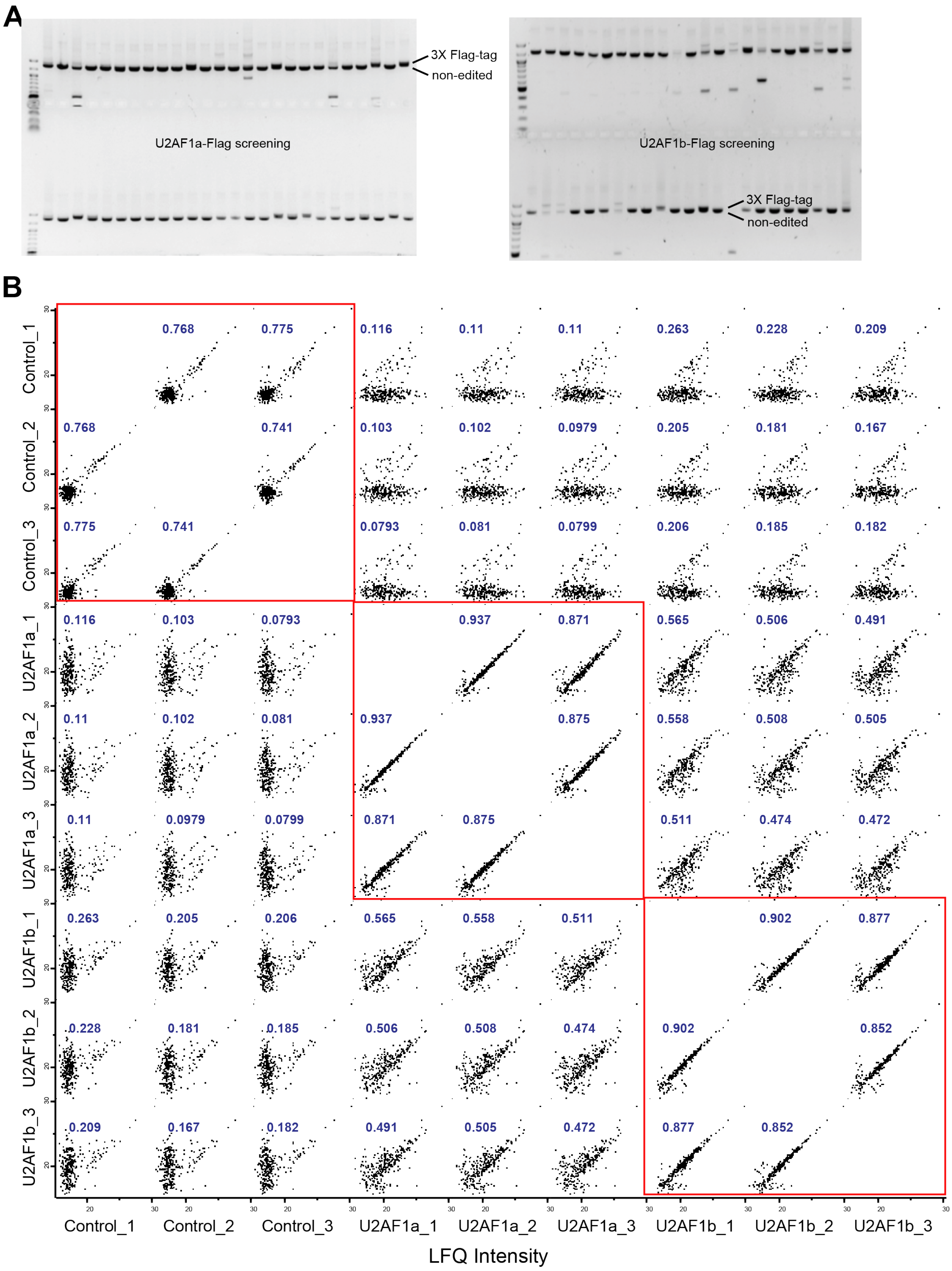

A
3X Flag-tag
non-edited
U2AF1a-Flag screening
U2AF1b-Flag screening
3X Flag-tag
non-edited
B
Control_1
Control_2
Control_3
U2AF1a_1
U2AF1a_2
U2AF1a_3
U2AF1b_1
U2AF1b_2
U2AF1b_3
0.768 0.775 0.116 0.11 0.11 0.263 0.228 0.209
0.768 0.741 0.103 0.102 0.0979 0.205 0.181 0.167
0.775 0.741 0.0793 0.081 0.0799 0.206 0.185 0.182
0.116 0.103 0.0793 0.937 0.871 0.565 0.506 0.491
0.11 0.102 0.081 0.937 0.875 0.558 0.508 0.505
0.11 0.0979 0.0799 0.871 0.875 0.511 0.474 0.472
0.263 0.205 0.206 0.565 0.558 0.511 0.902 0.877
0.228 0.181 0.185 0.506 0.508 0.474 0.902 0.852
0.209 0.167 0.182 0.491 0.505 0.472 0.877 0.852
LFQ Intensity

C

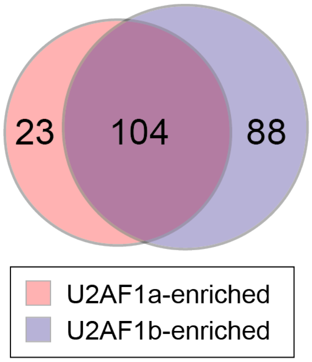

E

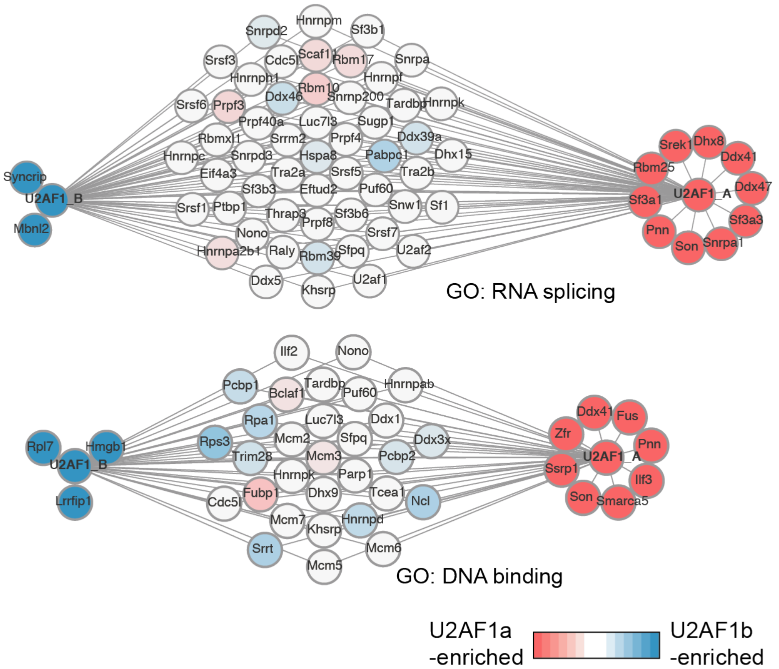

D

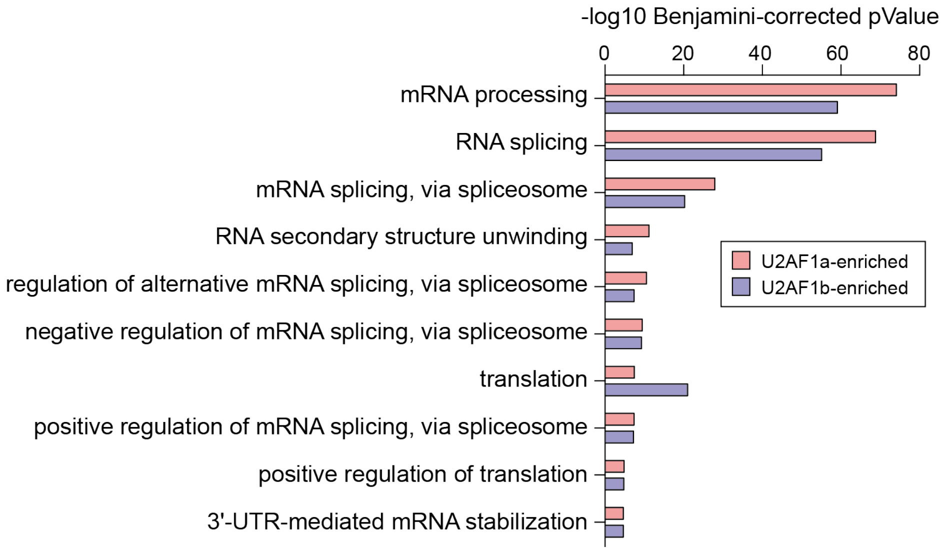

Supplementary Figure 4. (**A**) Screening of U2AF1a-Flag and U2AF1b-Flag cell lines after CRISPR/Cas9-initiated HR using synthesized gBlocks DNA. A slight shift caused by 3X-Flag tag insertion into the C-terminus of *U2af1* locus in U2af1**a**- and U2af1**b**-only cell lines was marked. (**B**) Multi-scatter plot illustrating the protein LFQ intensity and R^2 correlation among three sample repeats. Within technical replicates of each U2AF1 pull-down, the average R-

squared pair-wise correlation coefficients of the protein LFQ intensity profiles was 0.885, indicating high similarity. In contrast, the pair-wise comparison of the U2AF1 isoform-specific pulldowns had a lower average R-squared correlation coefficients (0.510) than the background protein correlation profile represented by the control Flag-IP samples using untagged $Tsc1^{-/-}$ cell line (0.761), indicating significant U2AF1 isoform-dependent difference in protein interactor abundance. (**C**) A summary of U2af1 isoform-specific interactomes. Overlapped and unique proteins identified by interactome studies are shown in a Venn Diagram. (**D**) GO-term analyses of U2AF1a- and U2AF1b-specific interactomes. (**E**) Interactomes of U2AF1a and U2AF1b in GO term RNA splicing and DNA binding are illustrated. Proteins colored in solid blue and red represent unique interactors of U2AF1b and U2AF1a, respectively.

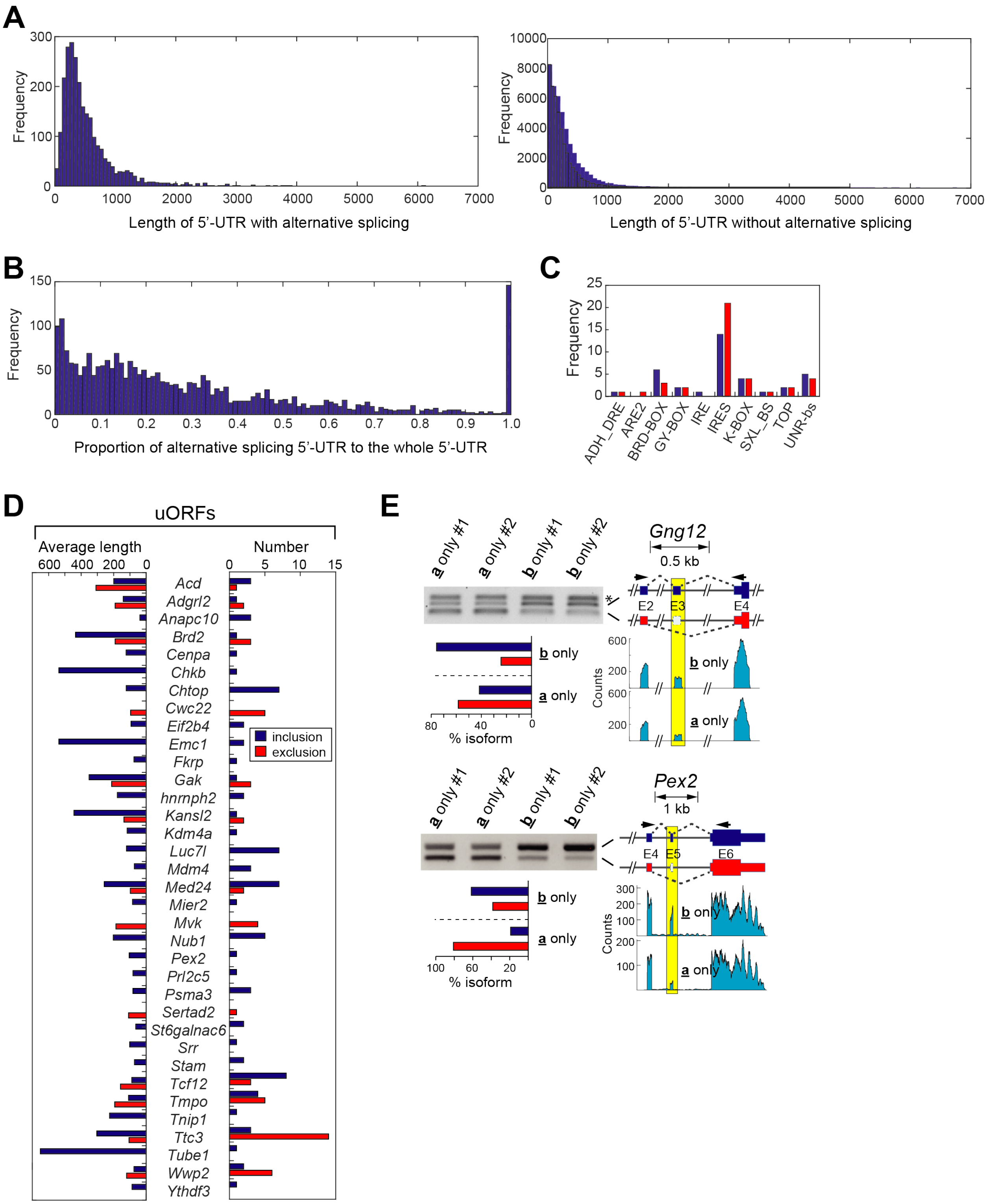
A
Frequency
Length of 5'-UTR with alternative splicing
Length of 5'-UTR without alternative splicing
B
Frequency
Proportion of alternative splicing 5'-UTR to the whole 5'-UTR
C
Frequency
ADH_DRE
ARE2
BRD-BOX
GY-BOX
IRE
IRES
K-BOX
SXL_BS
TOP
UNR-bs
D
uORFs
Average length
Number
Acd
Adgrl2
Anapc10
Brd2
Cenpa
Chkb
Chtop
Cwc22
Eif2b4
Emc1
Fkrp
Gak
hnrnph2
Kansl2
Kdm4a
Luc7l
Mdm4
Med24
Mier2
Mvk
Nub1
Pex2
Prl2c5
Psma3
Sertad2
St6galnac6
Srr
Stam
Tcf12
Tmpo
Tnip1
Ttc3
Tube1
Wwp2
Ythdf3
inclusion
exclusion
E
a only #1
a only #2
b only #1
b only #2
Gng12
0.5 kb
E2
E3
E4
b only
a only
% isoform
Counts
Pex2
1 kb
E4
E5
E6
b only
a only
% isoform
Counts

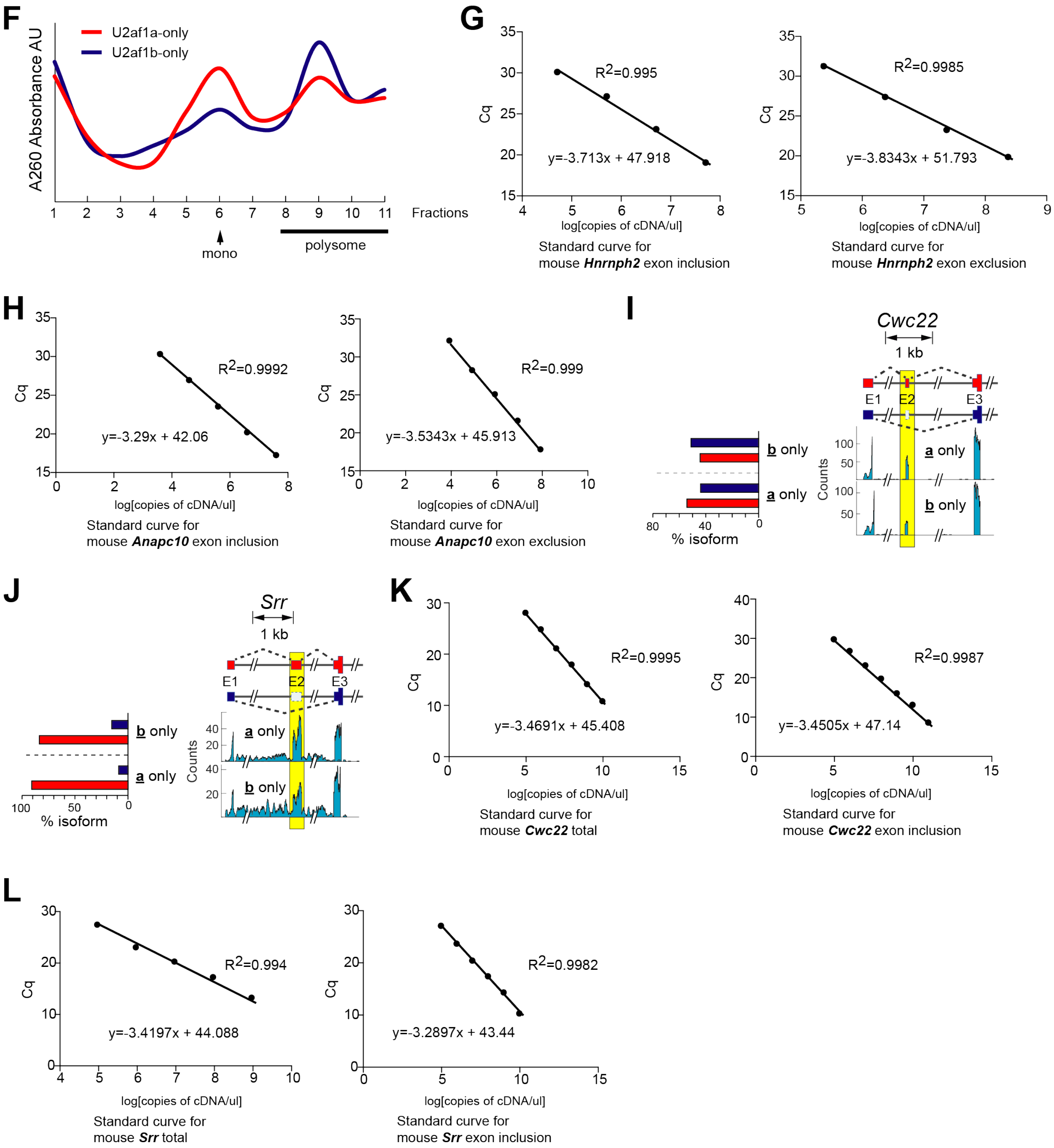
F
U2af1a-only
U2af1b-only
A260 Absorbance AU
1 2 3 4 5 6 7 8 9 10 11
Fractions
mono
polysome
G
R2=0.995
y=-3.713x + 47.918
Cq
log[copies of cDNA/ul]
Standard curve for
mouse Hnrnph2 exon inclusion
R2=0.9985
y=-3.8343x + 51.793
Cq
log[copies of cDNA/ul]
Standard curve for
mouse Hnrnph2 exon exclusion
H
R2=0.9992
y=-3.29x + 42.06
Cq
log[copies of cDNA/ul]
Standard curve for
mouse Anapc10 exon inclusion
R2=0.999
y=-3.5343x + 45.913
Cq
log[copies of cDNA/ul]
Standard curve for
mouse Anapc10 exon exclusion
I
Cwc22
1 kb
E1
E2
E3
b only
a only
% isoform
Counts
a only
b only
J
Srr
1 kb
E1
E2
E3
b only
a only
% isoform
Counts
a only
b only
K
R2=0.9995
y=-3.4691x + 45.408
Cq
log[copies of cDNA/ul]
Standard curve for
mouse Cwc22 total
R2=0.9987
y=-3.4505x + 47.14
Cq
log[copies of cDNA/ul]
Standard curve for
mouse Cwc22 exon inclusion
L
R2=0.994
y=-3.4197x + 44.088
Cq
log[copies of cDNA/ul]
Standard curve for
mouse Srr total
R2=0.9982
y=-3.2897x + 43.44
Cq
log[copies of cDNA/ul]
Standard curve for
mouse Srr exon inclusion

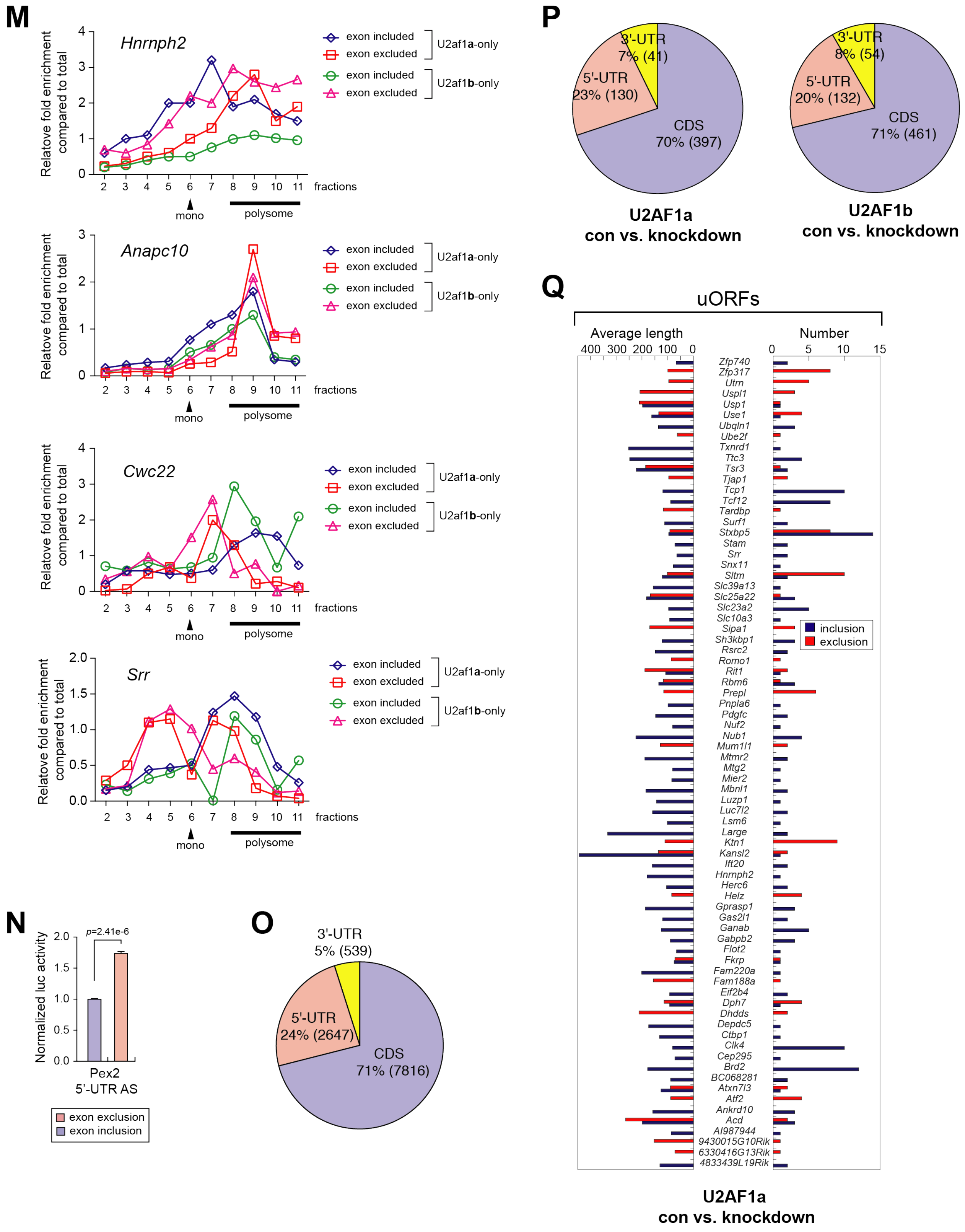
M
Hnrnph2
Anapc10
Cwc22
Srr
Relatove fold enrichment compared to total
fractions
mono
polysome
exon included
exon excluded
U2af1a-only
exon included
exon excluded
U2af1b-only
N
Normalized luc activity
p=2.41e-6
Pex2
5'-UTR AS
exon exclusion
exon inclusion
O
3'-UTR
5% (539)
5'-UTR
24% (2647)
CDS
71% (7816)
P
3'-UTR
7% (41)
5'-UTR
23% (130)
CDS
70% (397)
U2AF1a
con vs. knockdown
3'-UTR
8% (54)
5'-UTR
20% (132)
CDS
71% (461)
U2AF1b
con vs. knockdown
Q
uORFs
Average length
Number
inclusion
exclusion
Zfp740
Zfp317
Utrn
Uspl1
Usp1
Use1
Ubqln1
Ube2f
Txnrd1
Ttc3
Tsr3
Tjap1
Tcp1
Tcf12
Tardbp
Surf1
Stxbp5
Stam
Srr
Snx11
Sltm
Slc39a13
Slc25a22
Slc23a2
Slc10a3
Sipa1
Sh3kbp1
Rsrc2
Romo1
Rit1
Rbm6
Prepl
Pnpla6
Pdgfc
Nuf2
Nub1
Mum1l1
Mtmr2
Mtg2
Mier2
Mbnl1
Luzp1
Luc7l2
Lsm6
Large
Ktn1
Kansl2
Ift20
Hnrnph2
Herc6
Helz
Gprasp1
Gas2l1
Ganab
Gabpb2
Flot2
Fkrp
Fam220a
Fam188a
Eif2b4
Dph7
Dhdds
Depdc5
Ctbp1
Clk4
Cep295
Brd2
BC068281
Atxn7l3
Atf2
Ankrd10
Acd
AI987944
9430015G10Rik
6330416G13Rik
4833439L19Rik
U2AF1a
con vs. knockdown

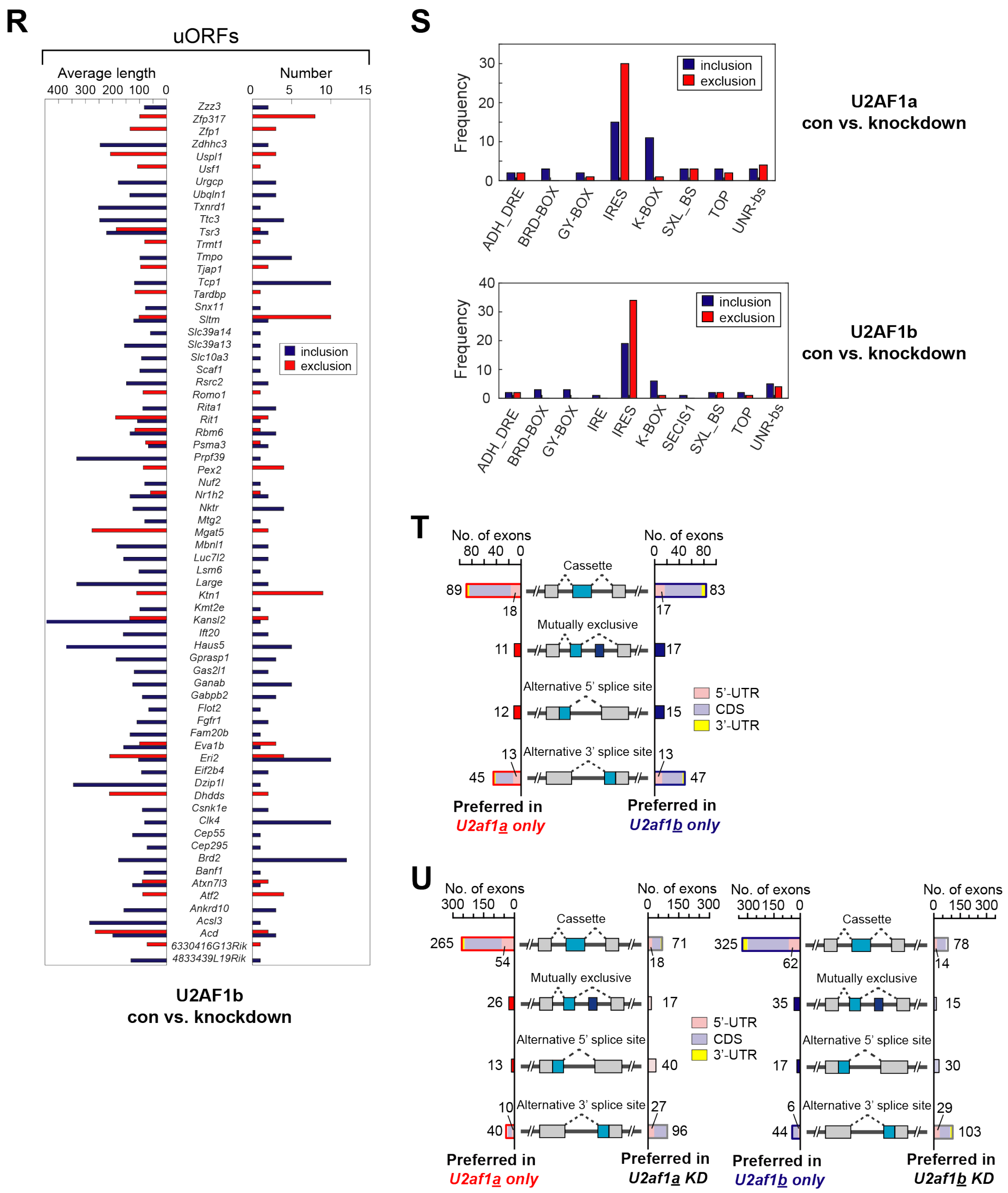

Supplementary Figure 5. (**A**) Length distribution of 5'-UTR with or without alternative splicing annotated in mm10 UCSC mouse genome annotation. (**B**) Proportion of alternative exon in 5'-UTRs with annotated alternative splicing events. The frequency of each portion was

displayed in the y-axis. (**C**) Rearrangement of known 5'-UTR regulatory elements by U2AF1a- and U2AF1b-mediated alternative splicing. Red boxes and blue boxes indicate the elements and frequencies changed by U2AF1a and U2AF1b, respectively. (**D**) Average length and number of uORFs changed by U2AF1a- and U2AF1b-mediated alternative splicing. Red boxes and blue boxes indicate the dynamics of uORFs by exon exclusion and inclusion, respectively. (**E**) Examples of 5'-UTR alternative splicing events in U2af1**a**- and **b**-only cells. RT-PCR and agarose gel electrophoresis were conducted to validate alternative splicing events. RNA-Seq read alignments and quantitation of alternative splicing events are shown. Arrows indicate the position of primer binding sites for RT-PCR analyses. Splicing isoforms and their quantitation are color-coded as illustrated; yellow boxes highlight the alternative exons. Asterisk denotes a non-specific PCR product. (**F**) Absorbance measurement of polysome fractionations. Fractions corresponding to monosme and polysomes are indicated. (**G, H**) Standard curves of qPCR assay for *Hnrnph2* (g) and *Anapc10* (h) 5'UTR alternative transcript isoforms. (**I, J**) Examples of 5'-UTR alternative splicing events in U2af1**a**- and **b**-only cells. RNA-Seq read alignments and quantitation of alternative splicing events are shown. Splicing isoforms and their quantitation are color-coded as illustrated; yellow boxes highlight the alternative exons. Asterisk denotes a non-specific PCR product. (**K, L**) Standard curves of qPCR assay for *Cwc22* (k) and *Srr* (l) 5'UTR alternative transcript isoforms. Due to technical difficulties, total- and long-specific qPCR primer sets were designed and used for absolute quantitation. (**M**) Data from polysome profiling analyses of Figure 5 (D-E) normalized to total input. Splicing isoforms are color-coded as denoted. **(N)** Luciferase assays on the effects of 5'-UTR alternative splicing events on translation efficiency. The 5'-UTRs including or excluding the alternative exon of *Pex2* were placed into the 5'-UTR of luciferase reporter. The fold-change of luciferase signals between the exon-included

and exon-excluded 5’-UTR reporter construct pair are shown in bar graphs. **(O, P)** Overall distribution of alternative splicing in the regions of mRNA was shown for (O) the mouse genome (mm10) and (P) U2AF1a control vs knockdown and U2AF1b control vs knockdown. **(Q, R)** Average length and number of uORFs changed by alternative splicing events in (Q) U2AF1a control vs knockdown and (R) U2AF1b control vs knockdown datasets. Red boxes and blue boxes indicate the dynamics of uORFs by exon exclusion and inclusion, respectively. **(S)** Rearrangement of known 5’-UTR regulatory elements by alternative splicing events in U2AF1a control vs knockdown and U2AF1b control vs knockdown datasets. Red boxes and blue boxes indicate the elements and frequencies changed by exon exclusion and inclusion, respectively. **(T)** Distribution of alternative splicing regions of mRNA in U2af1**a**- and U2af1**b**-only cell datasets. In each alternative splicing category, the distribution of regions is represented by color code. The number of alternative splicing events in cassette and alternative 3’-splice site categories was displayed. **(U)** Distribution of alternative splicing regions of mRNA in U2AF1a control vs knockdown and U2AF1b control vs knockdown datasets. In each alternative splicing category, the distribution of regions is represented by color code. The number of alternative splicing events in cassette and alternative 3’-splice site categories was displayed.
